# Morphological allometry constrains symmetric shape variation, but not asymmetry, of *Halimeda tuna* (Bryopsidales, Ulvophyceae) segments

**DOI:** 10.1371/journal.pone.0206492

**Published:** 2018-10-25

**Authors:** Jiri Neustupa, Yvonne Nemcova

**Affiliations:** Department of Botany, Faculty of Science, Charles University, Prague, Czech Republic; University of Illinois, UNITED STATES

## Abstract

Green algae of the genus *Halimeda* have modular siphonous thalli composed of multiple repeated segments. Morphological variation among the segments has been related to various environmental factors, which often jointly affect their size and shape. The segments are bilaterally symmetric, which means that their shape variation can be decomposed into the symmetric and asymmetric components. Asymmetric variation might reflect both environmental heterogeneity and developmental instability of morphogenetic processes during the development of segments. In the present study, we examined if segment shape in *H*. *tuna* is related to their size and if an allometric relationship can also be found with respect to their asymmetry. Relative contributions of directional and fluctuating asymmetry to the segment shape variation within individual plants were investigated at two close localities in the northern Adriatic Sea. A series of equidistant semilandmarks were set along the outline of the segments, and analyzed by geometric morphometrics using two parallel methods to optimize their final position. Symmetric variation was strongly constrained by allometry, which also explained differences between populations. Smaller segments were significantly more asymmetric, but the difference in asymmetry between populations could not be explained solely by this allometric relationship. These differences between populations might have been caused by variation in local environmental factors. We conclude that members of the genus *Halimeda* represent an intriguing model system for studies of morphometric symmetry and asymmetry of sessile marine organisms, including effects of allometric relationships and infraspecific variation in relation to environmental factors of the benthic coastal habitats.

## Introduction

Green algae of the genus *Halimeda* J.V.Lamouroux (Bryopsidales, Ulvophyceae) are typical by modular arrangement of their thalli, which are composed of multiple calcified segments joined by narrow non-calcified nodes [1]. Thus, like in vascular plants or many invertebrates, the architecture of their thalli is based on multiple repetitions of homologous structural motifs. While in arthropodes the entire body plans are based on segmentation into serially homologous structures [2], in vascular plants, these modular units are typically represented by leaves or flower parts [3, 4]. In bryopsidalean algae of the genus *Halimeda*, the modular units are flattened segments that are iterated during morphogenesis to compose a thallus formed by these repeated structural parts [1].

Segmented organisms made of multiple developmental modules represent important study systems for morphological plasticity research [2, 5, 6]. Different units within individuals are genetically identical and, thus, their shape differences reflect other sources of variation, such as position along the segmental series [5], enviromental heterogeneity [4, 7], or developmental noise [8]. While the within-individual shape variation of modular segments in streptophyte plants and invertebrates has recently been investigated in a number of studies [2, 5–7, 9], there are much less data on patterns of quantitative shape plasticity in the bryopsidalean green macroalgae, such as *Halimeda*. These organisms are typical by so called *translational symmetry*, i.e. the arrangement of parts that are repeated along the longitudinal body axis [8, 10]. In contrast to vascular plants, the thalli of *Halimeda* species are made of multinuclear, multiple-branched, and intertwined filaments lacking any cross cell walls. Hence, like in other Bryopsidales, the macroscopic thalli of *Halimeda* are not multicellular organisms but a single giant and multinuclear cell [1, 11]. Calcification of *Halimeda* thalli, which occurs in the interfilament spaces among the utricles that form the surface layer of the segments, makes them one of the most important organisms facilitating deposition of the precipitated CaCO_3_ in tropical and subtropical shelf ecosystems [1, 12]. All these characteristics make the members of the genus *Halimeda* an intriguing study system for an investigation of different geometric components of their segment shape variation and environmental or developmental factors driving this variation in a macroscopic organism lacking any cellular differentiation.

**Fig 1:**
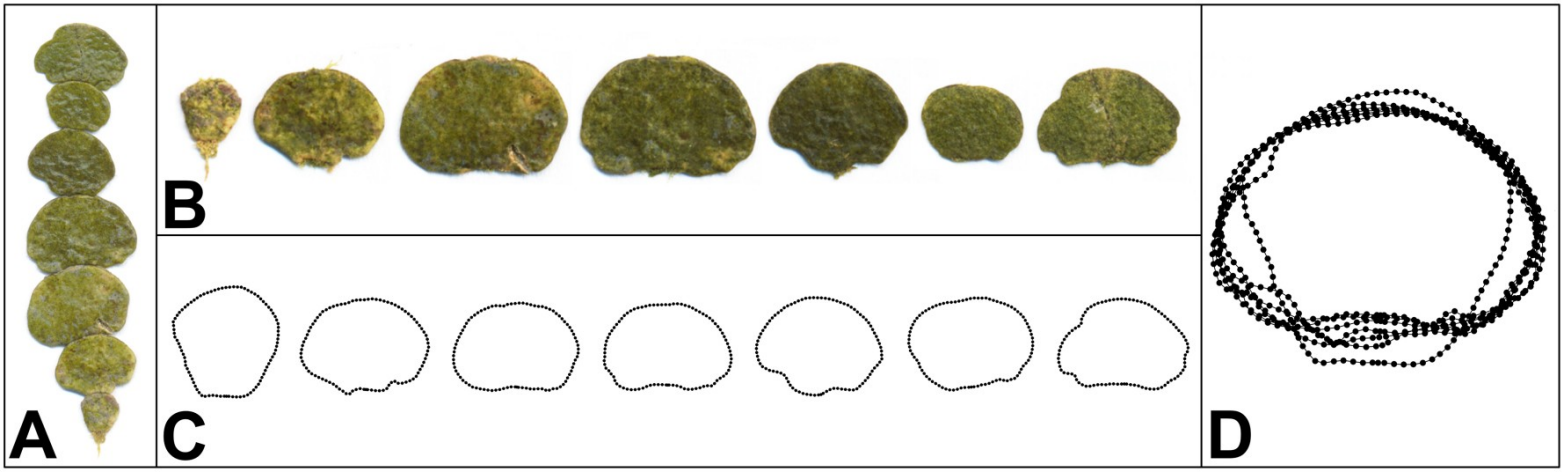
Branch of *Halimeda tuna* and the outline registration of segments. (A) Branch comprising seven consecutive segments. (B) Separated segments. (C) Semilandmarks digitized along the outlines. (D) Segments superimposed using generalised Procrustes analysis.

Currently, the genus comprises 45 species that differ in the anatomical structure of the filaments, as well as in shape, size and number of segments [13, 14]. Most of these species occur in the tropical sublittoral habitats that constitute the center of *Halimeda* evolutionary radiation. However, the type species of the genus, *Halimeda tuna* (J.Ellis & Solander) J.V.Lamouroux, was originally described from the Mediterranean [13]. This species is characterized by relatively large oval to reniform segments (Fig 1). In addition to being repeated many times within individual plants, the segments of *H*. *tuna*, as well as those of other members of the genus, are bilaterally symmetric. Thus, translational symmetry of the segments is accompanied by their bilateral object symmetry [15]. Therefore, morphological plasticity of these algae comprises both variation among the segments, as well as the components of morphological asymmetry between the segment halves.

Morphological plasticity among the segments can be conspicuous. Verbruggen and collaborators showed that "deviant segments" with shapes significantly deviating from taxonomic description of their respective species effectively prevent unambiguous morphometric classification of the segments into individual taxa [16]. Several studies also illustrated that variation of the segments is related to various environmental factors within the marine sublittoral habitats [17–19]. It has been shown that the size of *Halimeda* segments is often negatively correlated with high irradiation levels typical for shallow habitats close to the sea surface [20, 21]. This may lead to large individuals comprising relatively high number of large segments growing in less irradiated, deeper parts of the sublittoral. Conversely, photoinhibition, which occurs in *Halimeda* populations growing in shallow and highly irradiated locations in the upper sublittoral [22, 23], may be coupled with relatively small plants characterized by a compact habitus and small segments [17, 21]. These small segments of highly irradiated populations have also been reported as being relatively less calcified than those of populations thriving in shaded habitats [17]. The compact morphology of plants with small and mutually appressed segments has been interpreted as a mechanism to avoid photoinhibition due to the low exposure of individual segments to solar irradiation [21]. However, comparatively higher growth rates and larger dimensions of plants growing in deeper sublittoral habitats might also be related to locally higher nutrient concentrations in these areas [17]. The size of the segments and of the entire thalli can also be influenced by the mechanical disturbance caused by wave action [18, 24]. In fact, plants growing at exposed localities with strong and frequent wave action usually have reduced thalli with relatively small segments [18]. The variation observed among segments may also be allometrically related to their size. It has been shown that segments with deviant shapes are more frequent among those that are not fully calcified or occur in a basal position within thalli [16]. Interestingly, these deviant segments were also typically smaller than normal-shaped segments indicating that an allometric shape-to-size relationship may facilitate the morphological variation of the segments. However, an explicit analysis of the morphological allometry in shape of the *Halimeda* segments has not yet been conducted.

Translational series of the segments along a single branch is accompanied by their bilateral object symmetry [15, 25]. This means that they are symmetric *per se* and that their axis of symmetry divides them into two symmetric halves. Deviations from symmetry across this axis lead to the asymmetric variation of the segments. An ideal bilateral symmetry would be anatomically underlain by the synchronized trichotomic branching of medullary filaments in the developing segment [1]. However, the actual shapes of the segments usually more or less visibly deviate from ideal symmetry. Interestingly, *Oreotlos etor*, the leucosiid crab species that mimics *Halimeda* segments with its carapace, is also typical by subtle but distinct asymmetries in its outline shapes [26].

Morphological asymmetry of the segments composing individual plants can be side-oriented, i.e., one half of the segment can be different from the other half in some characteristic shape feature that is repeated along the entire *Halimeda* branch. Such pattern obviously results in an asymmetric mean shape of the segments forming that particular plant. This within-plant directional asymmetry (DA) is an attribute of a sample (i.e., the segments forming a single *Halimeda* branch). However, individual segments also have an additional asymmetry with respect to the mean shape established in the previous step. This fluctuating asymmetry (FA) is then an attribute of each individual segment within a *Halimeda* branch.

Separation of the bilateral asymmetry of the *Halimeda* segments into their directional and fluctuating components must be confined to individual branches because the segments are not differentiated along an anterior-posterior axis. This means that they do not have the front and back faces that would unambiguously align their left and right sides among different plants. It should be noted that this feature is typical for most algae and protists, but it is very rare or even lacking in bilateral animals and vascular plants [15]. A number of different microalgae are characterized by complex symmetric arrangement of their cellular parts, such as the lobes of Desmidiales or Hydrodictyaceae [27, 28] or frustules of pennate diatoms [29, 30]. However, due to side ambiguity, symmetry analysis of these structures often cannot involve the separation of the individual components of asymmetry. Thus, *Halimeda* thalli are unique among algae as their modular arrangement allows separating average asymmetry and individual asymmetric deviations of the segments at least at the level of individual plants.

Directional asymmetry, i.e., the average difference in shape of symmetric parts of a bilaterally symmetric structure, is usually genetically fixed in different animal model organisms [31–33]. In fact, geometric morphometric studies considering quantitative morphological symmetry have shown that most symmetric structures of animals and vascular plants have a subtle but clearly non-random DA in shape [15]. However, DA of multiple repeated symmetric structures at the level of individual specimens of plants and some sessile invertebrates might also be due to the spatially directed environmental heterogeneity in their immediate surroundings causing directionally asymmetric development [3, 4, 7]. Fluctuating asymmetry in shape, i.e. individual asymmetric deviations that can be detected after accounting for DA, has been widely used as an indicator of developmental instability, which has often been related to individual quality or increased environmental stress during their development [8, 15]. In marine organisms, increased FA has been frequently correlated with pollution-related environmental stress [34–36]. Thus, benthic marine algae, such as members of the genus *Halimeda*, may represent interesting model organisms for analyzing the effects of environment on phenotypic plasticity and developmental instability of their morphological structures. As sessile organisms, their shape variation could very well reflect the amount of environmental stress during segment morphogenesis, which encompasses a relatively short time span of two to three days before intense CaCO_3_ precipitation in the interutricular space makes their forms fixed. Variation among the segments of individual plants due to phenotypic plasticity could indicate the extent of their reaction norm, i.e., the range of phenotypes produced by a single genotype in environmentally variable conditions. FA of the segments would then denote the developmental instability caused by the environmental stress disturbing the symmetric morphogenesis of their left and right halves.

The present study evaluates the shape-to-size relation and morphological asymmetry of *H*. *tuna* segments using several morphometric and statistical procedures including Procrustes superimposition of semilandmarks, i.e., a series of consecutive points depicting the outline curve that is homologous among the studied objects [37, 38]. Due to the wide array of possible applications and straightforward combination of this kind of outline analysis with shape information derived from fixed landmarks, semilandmark-based studies have become popular in modern morphometrics [39]. The key feature of semilandmark analysis is that the position of individual points is solely defined by their order within the sequence of semilandmarks depicting the analyzed outline. However, the outlines spanned by semilandmarks can be superimposed using several different techniques. Although all of them remove the non-shape variation, such as different position, rotation, and size of the objects, they still lead to different residual distances among corresponding semilandmarks and, consequently, to different description of shape variation in analyzed datasets [38, 40]. One method of the semilandmark treatment involves their arrangement in equidistant positions along the outlines followed by the Procrustes superimposition [40]. Alternatively, semilandmarks can be slid iteratively along the tangents to the outline curve, assuring better correspondence of the curve with respect to the average configuration [38]. However, different criteria can be used for assessing this correspondence, such as the minimum thin-plate spline bending energy (BE) that is required to achieve deformation of the consensus curve to the analyzed specimen [37], or the minimum Procrustes distance (PD) between the outline points of a specimen and corresponding points of the consensus [41]. The effects of these different methods of semilandmark superimposition on the multivariate structure of shape data were compared in several recent studies [38, 39, 42], but their effects on the decomposition of symmetric and asymmetric components of shape variation have not yet been evaluated. Therefore, it is unclear to what extent would the choice of the sliding strategy affect the results concerning asymmetry of the segments. Consequently, the three methods of the semilandmark treatment were used in parallel in the present study and their effects on the decomposition of shape symmetry and asymmetry in *H*. *tuna* segments were compared using several multivariate Procrustes analysis of variance (ANOVA) models.

The geometric morphometric analyses were employed to answer the following questions and hypotheses: Is there any detectable average asymmetry (i.e., within-plant DA) between both halves of the segments at the level of individual *H*. *tuna* plants? If yes, how is it related to random asymmetric variation (FA), which reflects developmental instability of the morphogenetic processes? At the same time, we also test for the relation of different components of shape asymmetry to segment size. With these analyses, we aim to provide a new framework for morphometric research on the genus *Halimeda*, which could involve a suite of ecological and evolutionary topics related to morphological variation of these important primary producers and calcifiers of the marine benthic habitats.

## Material and methods

### Sampling

*H*. *tuna* thalli were sampled on September 2015 from two localities, assigned here as "A" and "B", set at the coast of Trieste Bay, Northern Adriatic, Slovenia (A—45° 35′ 37″ N, 13° 42′ 43″ E; B—45° 31′ 35″ N, 13° 35′ 09″ E). No specific permissions for sampling were required for the locations of the study as they are not located in any protected area and the study did not involve utilization of genetic resources in the sense of the EU Regulation no. 511/2014. In addition, the field studies did not involve any endangered or protected species. The access to the resources followed the due diligence steps according the above mentioned regulation.

These localities have analogous ecological conditions and they also host similar sublittoral algal communities [43, 44]. In the Adriatic Sea, *H*. *tuna* occurs as the sole *Halimeda* species and it is also the only native member of the genus occurring in the Mediterranean [45]. At both localities, *H*. *tuna* thalli were sampled in the upper sublittoral at 150–175 cm depth, in 2 × 2 m squares. The shore at both these localities is oriented towards the north. Population A grew at a flat bedrock bottom exposed to sunlight and irradiation of this locality was not significantly obstructed by any coastal structures, a prominent 25 meters high coastal cliff consisting of Eocene flysch sediments shaded the southern horizon for population growing at the locality B. This variation in coastal morphology might have caused lower irradiation levels between these two sites but these differences weren’t explicitly evaluated. Instead, the open sky proportion (OSP) at the sea surface level just above the sampling plots was quantified by an image analysis of two circular photographs taken on the sampling day by the camera equipped with the 8mm fisheye lens. These images were analysed with Gap Light Analyzer, ver. 2.0 [46] yielding the OSP values of 92.3% for the locality A and 63.5% for the locality B.

Individual branches were sampled to include all segments from the holdfast (which, however, was not included) up to the apical parts. On the same day, the segments were carefully separated, pressed on paper and scanned (Fig 1A and 1B). Non-calcified apical segments with unfinished growth and morphogenesis were omitted, as well as the occasional segments with fish bites. In total, 48 branches from separate plants from each population were analyzed and these 96 branches consisted of 982 segments.

### Digitization of landmarks

The outlines of the segments were captured by a single fixed landmark and 89 semilandmarks. The fixed landmark was placed onto the basal node of each segment, which is the starting point of its morphogenesis. All other points around the outline were set as the semilandmarks depicting its shape (Fig 1C and 1D). It should be noted that a fixed landmark cannot be placed onto the apical part of an outline where a new segment might have been initiated and developed, as the apical segments obviously lack any such point and, intercalary segments may have two or, ocassionally, even three segments initiated from their margins. The semilandmarks were digitized using the automated *background curves tool* of TpsDig, ver. 2.22. [47]. Their equidistant positions along the outlines were achieved using function *digit*.*curves* of the *geomorph* package, ver. 3.0.5 [48], in R, ver. 3.2.3 [49]. To assess measurement error, all objects were digitized twice. In the first digitization, outlines were registered clockwise, while in the second digitization they were registered counter clockwise and subsequently relabeled to match the order of the first digitization.

### Superimposition and treatment of semilandmarks

The resulting dataset, consisting of 982 × 2 = 1964 configurations (S1 Dataset), was subjected to three parallel generalized Procrustes analyses (GPAs) that differed in treatment applied to semilandmarks. First, semilandmarks were treated in the same way as fixed points and superimposed using GPA without any additional sliding along segment outlines. Thus, their position was solely determined by the equal Euclidean distances among them. Alternatively, an additional step was added into the superimposition algorithm that allowed semilandmarks to slide iteratively between their neighboring points along tangents to the respective curve. In this case, two parallel methods for determining the final position of semilandmarks were used. In the first option, their final position yielded the smoothest possible deformation of the whole configuration from the mean shape of the studied dataset, i.e. the position typical by the smallest possible BE between a configuration and the mean shape [37, 39]. When the PD criterion was used instead of BE, each semilandmark slid separately along the tangential line arriving at a position where PD between a particular configuration and the reference configuration is minimized [38, 41]. It should be noted that in this method each point slides separately. Therefore, in a dataset with considerable shape variation, which inevitably leads to relatively large differences between the consensus configuration and individual specimens, some semilandmarks may slide behind the position of their neighbors [38].

The effects of these three treatments on the shape of the segments was formally evaluated by three multivariate Procrustes ANOVA models. Each of these analyses decomposed shape variation into a dataset consisting of segment configurations superimposed with two different semilandmark treatment strategies. Thus, the datasets consisted of 1964 × 2 = 3928 configurations based on a) unslid equidistant semilandmarks and those slid by minimum BE criterion, b) unslid equidistant semilandmarks and those slid by minimum PD criterion and, c) semilandmarks slid by minimum PD and minimum BE criteria. A type I Procrustes ANOVA model fitted the variation spanned by shape differences among the different segments within each of these datasets. Then, it was used to quantify the effect of the superimposition strategy on the shape of each segment. The mean squares (MS) of this effect were evaluated against the MS of the digitization error in individual segments.

### Principal component analysis (PCA) and morphological allometry

For the analysis of symmetry and asymmetry, the configurations of segments were subjected to two symmetry transformations [8, 10], corresponding to the identity (i.e., the original configurations) and to the reflection of the landmark configurations across the vertical axis of bilateral symmetry. Landmarks in the reflected configurations were relabeled to ensure that their order was consistent with that in the original configuration. The fixed landmark (no. 1) and the sliding landmark no. 46 represented the axis of bilateral symmetry and, thus, their order remained the same in both original and reflected/relabeled configurations [25]. The aligned original and reflected/relabeled configurations were subjected to PCA, which yielded principal components (PCs) spanning either symmetric or asymmetric variation [50]. Configurations positioned on individual PCs at the margins of the ordination space, which represent the shape changes of the segments spanned by a particular morphological trend, were shown as thin-plate spline deformations of the consensus shape.

Morphological allometry of *H*. *tuna* segments was evaluated by multivariate regression of the Procrustes aligned shape data on their centroid size [51]. The resulting outlines of the segments, reconstructed by the regression model, which represented the smallest and the largest specimens within the size range of the studied dataset, were illustrated as deformation grids from the reference form using the thin-plate spline function. The overall fit of the multivariate regression model was evaluated using Wilks’ λ and its significance was assessed by permutation tests with 999 random repetitions.

### Multivariate nested Procrustes ANOVA

The proportion of symmetric variation in segment shape that could be apportioned to individual levels of the sampling design, such as locality, plants, segments, asymmetric effects, and the digitisation error, were evaluated by multivariate non-parametric Procrustes ANOVA. The analysis was based on the matrix of tangent PDs among configurations, which was partitioned across the different sources of variation by fitting a linear model. Each segment entered the analysis in four separate instances, because there were two separate digitizations and it was represented by the original configuration, and by its reflected and relabeled copy [15]. Data were sampled in a nested structure that was reflected by the Procrustes ANOVA model. First, the symmetric variation in segment shape spanned by the difference between populations was quantified. Second, the symmetrized segment shapes typical for individual plants were evaluated. Then, the model tested for the symmetric variation among segments within individual plants. Finally, the two asymmetric effects nested within the level of individual plants (due to side ambiguity in segments from different plants) were evaluated: DA (the "side" effect) was represented by the average asymmetry between left and right halves of the segments and FA evaluated the random asymmetric fluctuations around the asymmetric mean. Measurement error was expressed as the sum of squares (SS) representing the difference between two independent digitizations of the outlines.

The nested structure of the data required appropriate construction of the F-ratios in the Procrustes ANOVA model (S1 Table). Thus, the MS of the main effect of "locality" were divided by the MS of the nested factor, i.e. individual plants. Likewise, the MS of plants were divided by the MS of the segments to yield the F-ratio for the "plant" factor. Within the level of individual plants the model evaluated the main effects of the symmetric variation among segments and their average asymmetry (i.e., DA). The F-ratios for these effects were acquired by dividing their MS by MS of their interaction, which represented FA [25]. The permutation strategy also reflected the nested structure of the data. Thus, the *p*-value for the effect of "locality" was assessed by randomizing the nearest subordinate nested factor (i.e., plants). Likewise, the *p*-value for plants was based on permutations of the segments. The *p*-values for symmetric variation among the segments, as well as their DA, were based on permutations of their original and reflected/relabeled copies within plants. Variation spanned by the FA of segments within plants was tested against the measurement error. All these analyses were based on 999 permutations. Morphological allometry might influence the structure of the variation that is apportioned to individual factors in the Procrustes ANOVA model. Therefore, we conducted a parallel analysis that accounted for the effect of centroid size on the shape variation of the segments before evaluating the nested factors.

In parallel to the overall decomposition of the shape variation across the different sources we also conducted a series of Procrustes ANOVA analyses for individual plants. In these analyses, each plant was taken separately, and individual segments were represented by their original and reflected/relabeled configurations. Thus, these analyses led to the decomposition of symmetric variation, DA, and FA in each of the 96 analyzed plants. Given the relatively low number of segments in plants, these analyses were not intended for significance testing of individual factors. Rather, we used them for comparing the contribution of these factors to shape variation within plants at both localities. While higher values for symmetric variation would indicate increased plasticity among the segments, higher FA could be attributed to increased levels of developmental instability during their development. For this comparison, we used the MS and R^2^ values yielded by the Procrustes ANOVA models. While MS values represent an estimate of the variance spanned by a particular factor in the model, R^2^ values depict the relative contribution of a factor to the overall fit. Regarding *Halimeda* plants, this means that higher MS values for a factor representing segments’ symmetry or any of the asymmetric components of their shape variation need not be accompanied by an increased R^2^ value if this factor is not relatively more important than other sources of variation in the Procrustes ANOVA model for a particular plant.

The MS and R^2^ values for symmetry, DA, and FA resulting from the Procrustes ANOVA analyses of the 96 *H*. *tuna* plants were used for formal comparison of the localities based on the 95% confidence interval of the difference in their medians. This confidence interval was created by bootstrapping original values for individual plants. If the 95% confidence interval included zero, we concluded that there was no significant difference between the medians of sets [52]. This bootstrap (BS) analysis was also used for comparing centroid size values and the number of segments forming individual plants between both populations. Finally, the MS values for the three components of variation within plants, i.e. symmetric variation among segments, their DA, and FA, were compared by linear correlation analyses. These correlations showed whether increased variation in one component was usually accompanied by a similar increase in other components, or whether these three components were mutually independent in their amounts of variation within the plants.

### Total shape asymmetry

Contrary to symmetric variation, the relationship between allometry and shape asymmetry cannot be evaluated based on the Procrustes ANOVA models described above. In structures with object symmetry, size obviously remains the same in both original and reflected/relabeled configurations. Therefore, allometric effects on asymmetry cannot be evaluated by accounting for size in the matrix of Procrustes distances. However, allometric effects in shape asymmetry can be evaluated based on the asymmetric PCs yielded by the PCA of both original and reflected/relabeled configurations. The scores of each original configuration on the asymmetric axes mirror the scores of their reflected/relabeled counterparts [10]. Therefore, even in structures with side ambiguity as *Halimeda* segments, we can use the sum of the Euclidean distances of PC scores from all the original or the reflected/relabeled configurations as a quantitative measure of total shape asymmetry. This measure can then be used as the dependent variable, which is decomposed into different sources of variation in a univariate nested ANOVA model corresponding to prior multivariate analyses of the overall patterns of the shape variation. Likewise, this analysis also included a version that accounted for the allometric effect of centroid size on total shape asymmetry of the segments before evaluating the nested factors.

All the morphometric analyses were conducted using the functions included in the packages geomorph, ver. 3.0.0 [48], shapes, ver. 1.1–11 [53], and BiodiversityR, ver. 2.8–0 [54], as well as the core functions of R, ver. 3.2.3 [49]. In addition, PAST, ver. 2.17c [55], was used for data handling and linear correlation analyses. Finally, multivariate regression and PCA were conducted in TPSRegr, ver. 1.31, and TpsRelw, ver. 1.67, respectively [47].

## Results

### Superimposition of semilandmarks

PDs were relatively large irrespective of the type of semilandmark treatment. Mean PD was 0.109 in the unslid dataset, 0.124 in configurations slid to minimium BE and 0.090 in those slid to minimum PD. The maximum PDs among the most divergent specimen and the reference configuration were 0.412, 0.439, and 0.324 for the unslid, minimum BE, and minimum PD datasets, respectively.

**Fig 2:**
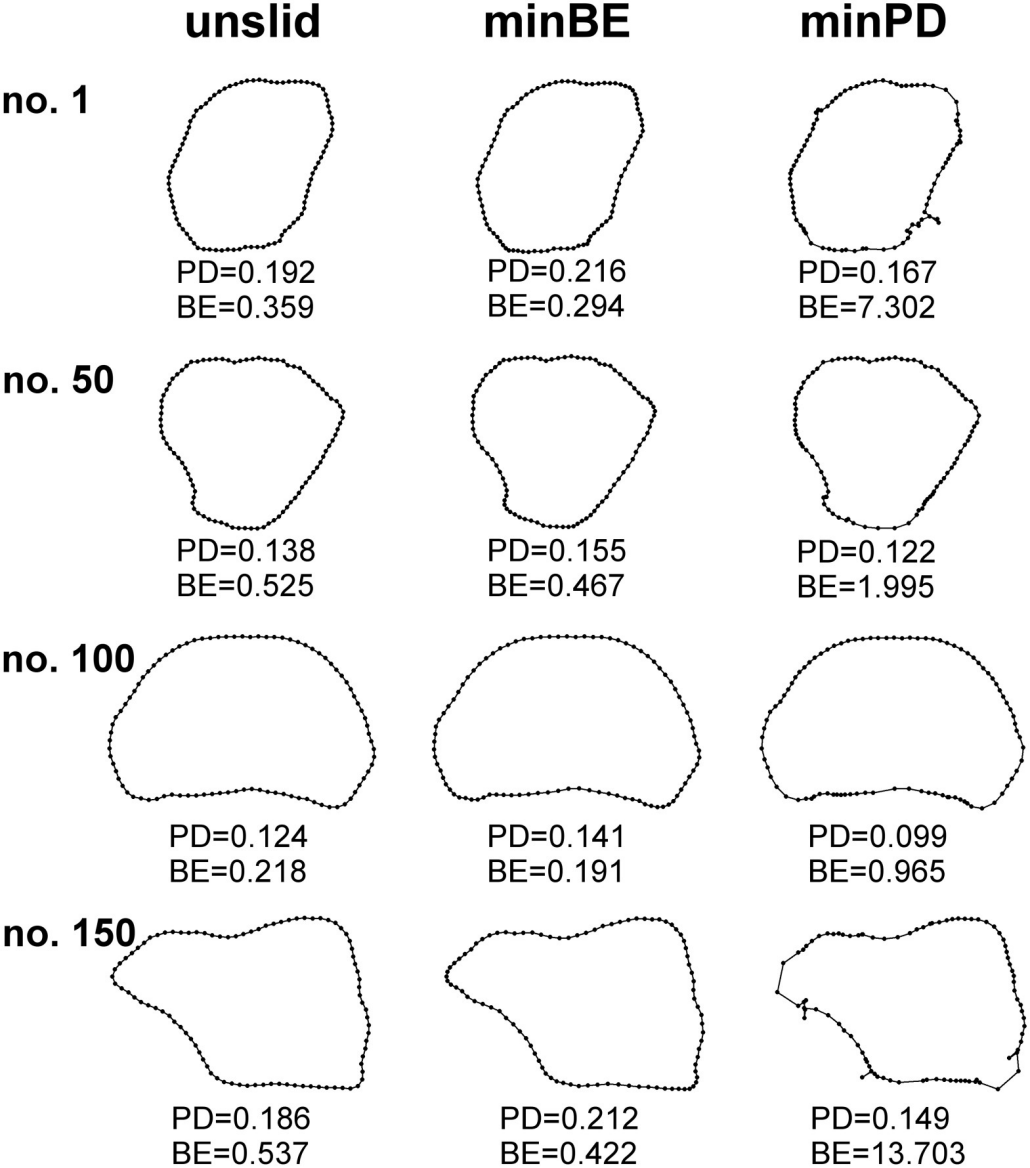
Outlines of four *H*. *tuna* segments from the overall dataset registered by the three parallel strategies of semilandmark treatment. Numbers refer to the order of segments in the studied dataset. unslid = equidistant semilandmarks without any additional sliding; minBE = minimum bending energy; minPD = minimum Procrustes distance. PD = Procrustes distance of the actual segment to the consensus configuration. BE = Thin-plate spline bending energy from the consensus configuration to the depicted segment.

The GPA that included additional sliding of the equidistant semilandmarks based on the minimum BE between each segment and the mean configuration produced shapes closely similar to those yielded by the GPA that lacked any additional sliding steps. The effect of semilandmark sliding using the minimum BE criterion on the resulting shape configuration of individual segments was approximately the same as the measurement error arising from the repeated digitisation of the outline curves (S2 Table). In addition, this sliding strategy did not visibly distort the shapes of the outlines (Fig 2).

Conversely, configurations in which semilandmarks were slid based on minimum PD were considerably different from the other two datasets. Using this method, the effect of sliding on the shape of the segments was about 4.0 and 5.8 times higher than the digitization error (S2 Table). Semilandmark sliding with minimum PD criterion was clearly achieved at a cost of apparent distortions of the outline shapes (Fig 2). Such dataset is clearly unsuitable for an analysis of the supposedly subtle shape deviations between the symmetric halves of the segments. Thus, further analyses were only based on the datasets yielded by the superimposition of the semilandmarks slid along their tangential lines using the minimum BE criterion and the unslid equidistant points along the outlines.

### Morphological allometry and PCA

**Fig 3:**
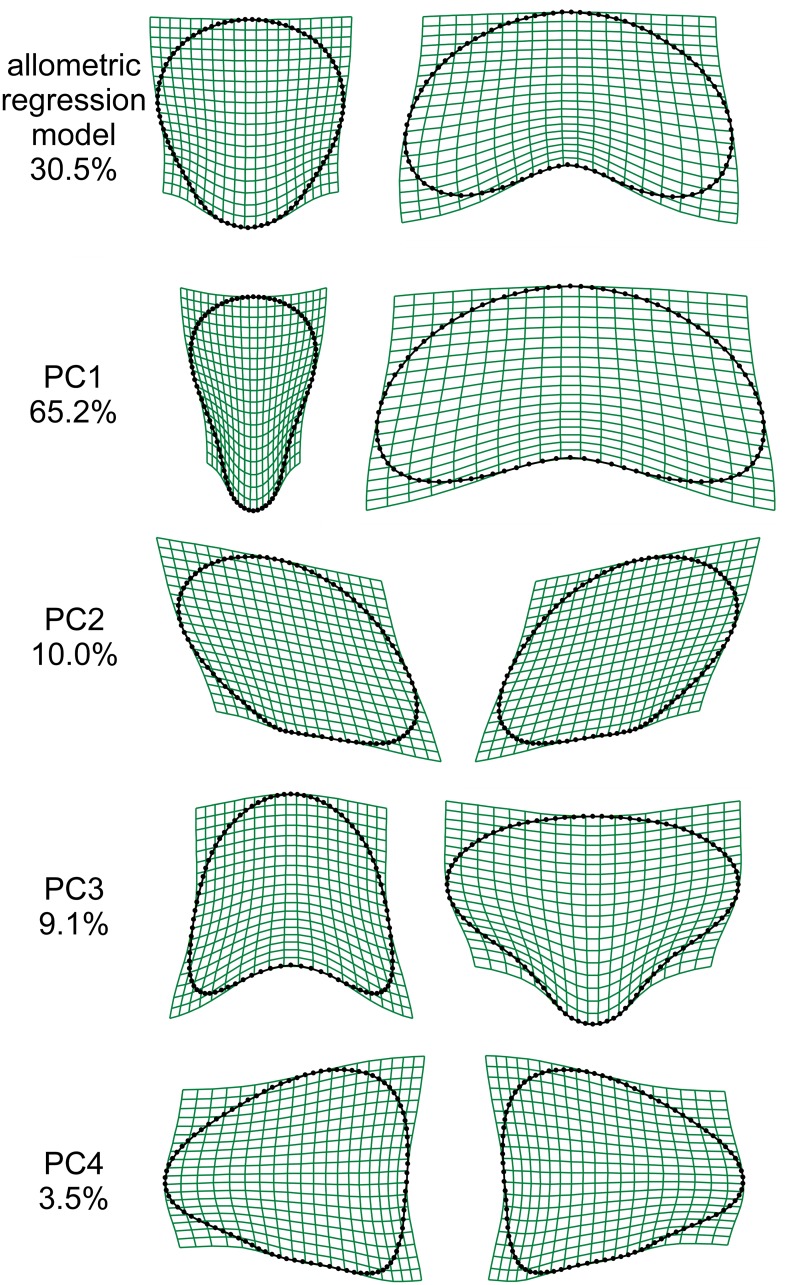
Thin-plate splines illustrating the multivariate allometric regression model and principal component analysis. Analyses were based on generalized Procrustes analysis including the sliding of semilandmarks using minimum bending energy criterion. Thin-plate splines depict the deformation from the consensus configuration to marginal positions on individual axes.

Although analysed branches from both populations had the same median number of segments (9.5), those from the population A were distinctly smaller with median centroid size about 18% lower than in the population B. This difference was highly significant in the bootstrap tests as none of the 999 random BS repetitions yielded a lower median centroid size in population B than in population A. The multivariate regression of the Procrustes coordinates on centroid size revealed a considerable morphological allometry in dataset. The allometric multivariate regression model explained 29.1% of the total variation in segment shape in the minimum BE dataset and 30.6% in the unslid Procrustes dataset (S3 Table). The morphological pattern associated with the allometric line differentiated small segments with an inversely conic shape from large segments with typical wide bean-like shapes (Fig 3). This morphological dynamics was largely reflected in the first principal component (PC1) yielded by PCA of the original and reflected/relabelled configurations (Fig 3), which explained 65.2% of the total variation and described the single most prominent pattern of the symmetric variation among the segments. Their shape along PC1 varied from inversely conic with distinctly elongated basal parts to wide bean-shaped segments similar to those shown by large segments in the allometric multivariate regression model. The second most important PC associated with symmetric variation, i.e., PC3, spanned 9.1% of the variation in the morphospace, and illustrated the difference between segments with distinct basal stalks and flat apical parts and segments with convex basal portions and rounded apical outlines. PC2 and PC4 were the most important axes that described morphological asymmetry. While PC2 describing 10.0% of the variation and was associated with the left-right leaning of the upper halves of the segments, PC4, describing 3.5% of the variation, spanned asymmetry pattern leading to segments with distinct left-right protrusion in the equatorial part of their outlines. The singular values and proportions of variation described by the first four principal components yielded by the PCA based on the unslid equidistant semilandmarks slightly differed from the values described above for the semilandmarks slid by the minimum BE criterion (S3 Table). However, PCs resulting from this analysis described virtually identical morphological patterns in the shape variation of the segments.

### Total shape asymmetry

Total shape asymmetry was obtained by adding the Euclidean distances of the PC scores of individual objects on all asymmetric axes yielded by the PCA of the original and reflected/re-labelled configurations. Nested ANOVA results showed that the degree of shape asymmetry of the segments clearly differed between populations A and B (Table 1). As both populations also differed in mean segment size, this shape difference might have been caused by an allometric size-to-asymmetry relationship. However, the subsequent ANOVA model that accounted for the effect of size on the total asymmetry before evaluating the nested factors showed that populations were still considerably different.

**Table 1:** Nested ANOVA models for total shape asymmetry of *H*. *tuna* segments.

**Minimum BE criterion**					
**Source of variation**	**df**	**SS**	**MS**	**F**	**p**
locality	1	0.0785	0.0785	27.984	0.001
plant (locality)	94	0.2637	0.0028	1.906	0.001
segment (plant)	886	1.3040	0.0015	4.822	0.001
measurement error	982	0.1122	0.0001		
total	1963	1.7584			
**Minimum BE criterion, accounting for allometry**					
**Source of variation**	**df**	**SS**	**MS**	**F**	**p**
centroid size	1	0.2124	0.2124	145.315	0.001
locality	1	0.0347	0.0347	15.093	0.001
plant (locality)	94	0.2159	0.0023	1.719	0.001
segment (plant)	886	1.1835	0.0013	3.059	0.001
measurement error	981	0.1119	0.0001		
total	1963	1.7584			
**Unslid semilandmarks**					
**Source of variation**	**df**	**SS**	**MS**	**F**	**p**
locality	1	0.0698	0.0698	30.041	0.001
plant (locality)	94	0.2185	0.0023	1.915	0.001
segment (plant)	886	1.0755	0.0012	5.023	0.001
measurement error	982	0.0834	0.0001		
total	1963	1.4472			
**Unslid semilandmarks, accounting for allometry**					
**Source of variation**	**df**	**SS**	**MS**	**F**	**p**
centroid size	1	0.1775	0.1775	146.794	0.001
locality	1	0.0319	0.0319	16.984	0.001
plant (locality)	94	0.1763	0.0019	1.699	0.001
segment (plant)	886	0.9783	0.0011	3.119	0.001
measurement error	981	0.0832	0.0001		
total	1963	1.4472			

BE = bending energy; df = degrees of freedom; SS = sum of squares; MS = mean squares.

Likewise, segments averaged at the level of individual plants, and segments within plants significantly differed in terms of total shape asymmetry (Table 1). These patterns did not change much when centroid size was accounted for, although centroid size alone was significantly correlated with total shape asymmetry with Pearson’s *r* = -0.35, which spanned 12.04% of the variation in asymmetry of segments.

The dataset comprising unslid configurations yielded similar patterns to those of configurations slid with the minimum BE criterion (Table 1). The total SS were about 18% lower in the unslid dataset than in configurations slid with the minimum BE criterion. However, F-ratios for individual effects were closely similar between analyses based on both superimposition strategies.

### Decomposition of the overall shape variation into different sources

The multivariate Procrustes ANOVA model for semilandmarks slid with the minimum BE criterion revealed that the segments composing plants from the two *H*, *tuna* populations significantly differed in their average symmetric shape variation (Table 2). In addition, symmetric variation of the segments averaged at the level of individual plants was significantly higher than that expected by chance. Within plants, segments strongly differed in their symmetrized shapes and a subtle but statistically significant DA was also recovered (*p =* 0.024). Finally, the FA of segment shape at the levels of individual plants was strongly significant against the digitization error (Table 2).

**Table 2:** Multivariate nested Procrustes ANOVA models for segment shape data. Factor “side” relates to DA and “segment×side” to FA, both nested within the plants.

**Minimum BE criterion**					
**Source of variation**	**df**	**SS**	**MS**	**F**	**p**
locality	1	0.948	0.9478	10.044	0.001
plant (locality)	94	8.871	0.0944	1.547	0.001
segment (plant)	886	54.041	0.0609	4.822	0.001
side (plant)	96	1.525	0.0159	1.256	0.024
segment×side (plant)	886	11.207	0.0127	10.820	0.001
measurement error	1964	2.361	0.0012		
total	3927	78.952			
**Minimum BE criterion, accounting for allometry**					
**Source of variation**	**df**	**SS**	**MS**	**F**	**p**
centroid size	1	22.635	22.635	823.407	0.001
locality	1	0.073	0.0730	0.997	0.322
plant (locality)	94	6.880	0.0732	1.892	0.001
segment (plant)	886	34.277	0.0387	3.059	0.001
side (plant)	96	1.525	0.0159	1.256	0.028
segment×side (plant)	886	11.207	0.0126	10.735	0.001
measurement error	1963	2.354	0.0012		
total	3927	78.952			
**Unslid semilandmarks**					
**Source of variation**	**df**	**SS**	**MS**	**F**	**p**
locality	1	0.847	0.8470	11.246	0.001
plant (locality)	94	7.080	0.0753	1.554	0.001
segment (plant)	886	42.938	0.0485	5.023	0.001
side (plant)	96	1.125	0.0117	1.214	0.048
segment×side (plant)	886	8.549	0.0097	10.820	0.001
measurement error	1964	1.429	0.0007		
total	3927	61.968			
**Unslid semilandmarks, accounting for allometry**					
**Source of variation**	**df**	**SS**	**MS**	**F**	**p**
centroid size	1	18.791	18.791	883.492	0.001
locality	1	0.063	0.0633	1.113	0.268
plant (locality)	94	5.349	0.0569	1.891	0.001
segment (plant)	886	26.664	0.0301	3.119	0.001
side (plant)	96	1.125	0.0117	1.214	0.032
segment×side (plant)	886	8.549	0.0096	10.735	0.001
measurement error	1963	1.427	0.0007		
total	3927	61.968			

BE = bending energy; df = degrees of freedom; SS = sum of squares; MS = mean squares.

The Procrustes ANOVA model designed to account for allometry in the shape of the segments, before evaluating the non-allometric variation into different sources, revealed that shape differences between populations disappeared when the size-related variation was removed (Table 2). However, plants still significantly differed within the localities regarding the shape of their segments. The symmetric variation among segments within individual plants was still significant, although with a lower F-ratio than in the Procrustes ANOVA model based on the original Procrustes coordinates. By definition of object symmetry, the asymmetric components within plants remained unchanged after removing the allometric variation.

The analyses of the unslid Procrustes aligned equidistant points produced similar relative proportions of variation spanned by individual effects. Populations were significantly different according to the nested Procrustes ANOVA model, but this difference once again proved to be allometrically determined by size variation of the segments. Conversely, symmetric variation in segment shape averaged at the level of individual plants and in segments within plants was strongly significant, regardless of prior accounting for the effect of morphological allometry. The DA within plants was slightly lower in unslid configurations than in configurations slid based on minimum BE, and the F-ratio of 1.214 yielded by comparison of the MS for DA against those for FA was only slightly significant in the permutation tests with respect to the customary 0.05 level. However, FA was highly significant when compared to the measurement error.

### Shape variation at the level of individual plants

**Fig 4:**
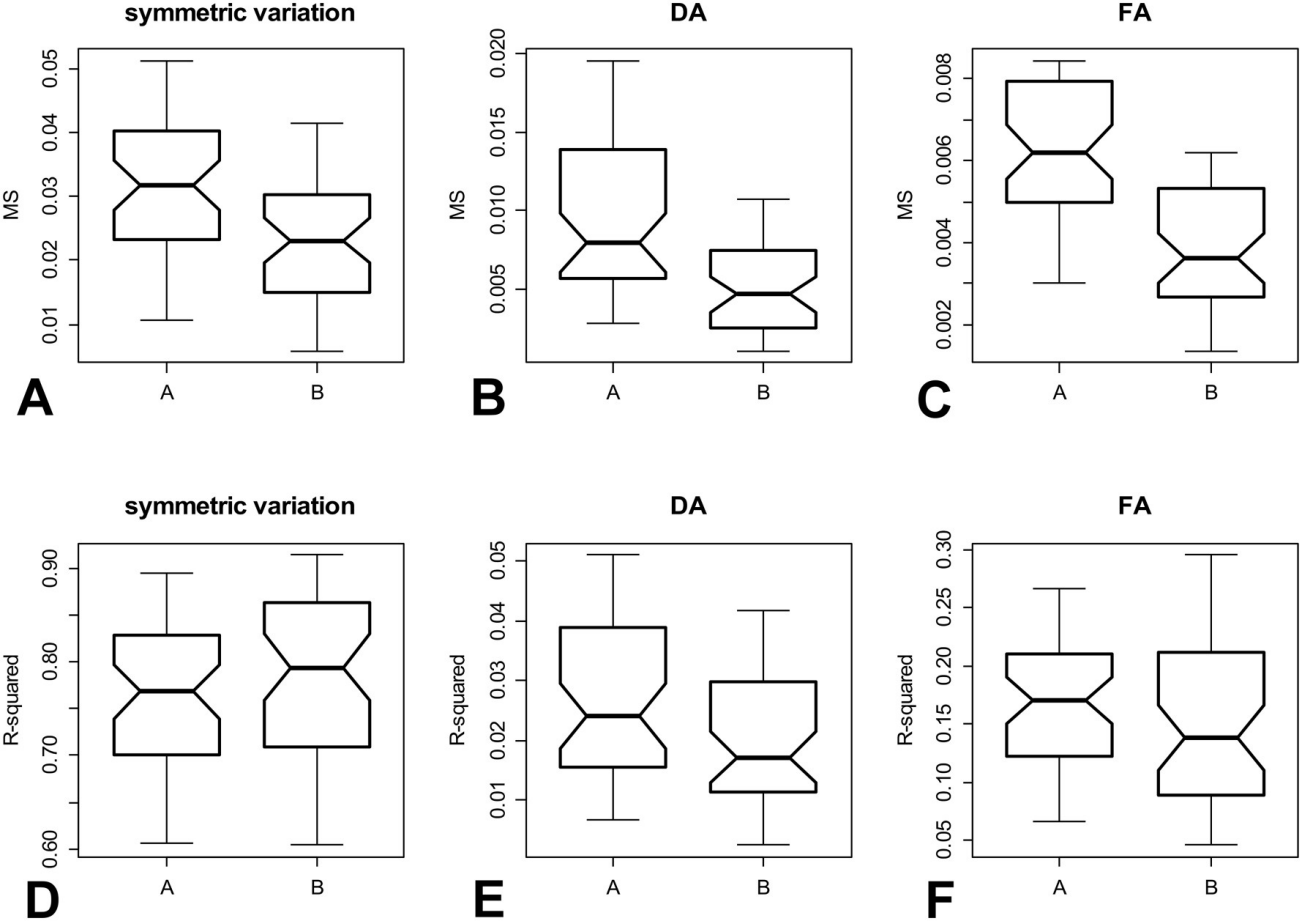
Comparison of the MS and R^2^ values yielded by the Procrustes ANOVA models of shape variation within plants at both populations. (A) MS values for symmetric variation depicting the differences in shape among the segments. (B) MS values for variation spanned by directional asymmetry. (C) MS values for fluctuating asymmetry. (D) R^2^ values for symmetric variation. (E) R^2^ values for directional asymmetry. (F) R^2^ values for fluctuating asymmetry.

The Procrustes ANOVA models for individual plants based on semilandmark configurations that included sliding with minimum BE yielded MS and R^2^ values for symmetric variation and both asymmetric components (S4 Table). The MS values obtained for the three components of the variation were significantly higher in plants sampled at the locality A, than in the population B (Fig 4, Table 3). This increased variation in one of the studied populations suggested that the three components of shape variation might be highly correlated within plants. However, this proved to be only partly supported by the linear correlation analyses performed for the MS values of the individual components of variation yielded by the Procrustes ANOVA runs for individual plants. Pairwise correlations among symmetric variation, DA, and FA were significant, but with rather modest Pearson’s *r* values ranging from 0.37 to 0.57, indicating that the MS values of individual shape components in individual plants were only slightly related (S5 Table).

**Table 3:** Results of bootstrap tests for the difference in components of segment shape variation between populations represented by configurations slid by the minimum BE criterion.

Factor	median (population A)	median (population B)	range of BS estimates for the difference between medians	95% CI for the difference between medians	*p*-value of BS estimates
Symmetric variation (MS)	0.0318	0.0231	-0.00014 ↔ 0.0147	0.0045 ↔ 0.0147	0.002
Symmetric variation (R^2^)	0.7688	0.7942	-0.0850 ↔ 0.0307	-0.0640 ↔ 0.0307	0.111
Directional asymmetry (MS)	0.0079	0.0046	0.00019 ↔ 0.0074	0.0018 ↔ 0.0074	0.001
Directional asymmetry (R^2^)	0.0241	0.0172	-0.0057 ↔ 0.0201	0.0018 ↔ 0.0201	0.017
Fluctuating asymmetry (MS)	0.0062	0.0036	0.00066 ↔ 0.0040	0.0015 ↔ 0.0040	0.001
Fluctuating asymmetry (R^2^)	0.1707	0.1385	-0.0428 ↔ 0.0858	-0.0067 ↔ 0.0858	0.075

BE = bending energy; BS = bootstrap; CI = confidence interval; MS = mean squares; R^2^ = coefficient of determination.

Asymmetric components also spanned a higher share of the total variation in plants from the population A (Fig 4B, 4C, 4E and 4F). The R^2^ values for DA were significantly higher in plants from this population, indicating that this asymmetric component was more represented in the shape variation of the segments composing these plants than the remaining components. However, differences in the median values of the R^2^ for FA were not significantly different from zero in the BS tests that yielded a *p*-value slightly above the conventional 0.05 level (Table 3). Conversely, symmetric variability among segments composed a slightly higher share of the total shape variation in plants from the population B (Fig 4D). However, differences in the median R^2^ values for this component of the shape variation also did not significantly differ from zero in the BS tests (Table 3).

Results of the analyses conducted with unslid equidistant Procrustes aligned semilandmarks were almost identical to that performed for semilandmark sliding using the minimum BE criterion (Table 4, S6 Table). Interestingly, the MS for individual components were consistently lower in the analyses of the unslid data than in those slid by minimum BE. However, differences among components of symmetry and asymmetry between populations remained unchanged.

**Table 4:** Results of bootstrap tests for the difference in components of segment shape variation between populations represented by configurations with unslid equidistant semilandmarks.

Factor	median (population A)	median (population B)	range of BS estimates for the difference between medians	95% CI for the difference between medians	*p*-value of BS estimates
Symmetric variation (MS)	0.0253	0.0177	0.00029 ↔ 0.01362	0.00418 ↔ 0.01362	0.001
Symmetric variation (R^2^)	0.7746	0.8128	-0.0314 ↔ 0.1073	0.0038 ↔ 0.1073	0.031
Directional asymmetry (MS)	0.0045	0.0027	-0.00077 ↔ 0.00499	0.00055 ↔ 0.00499	0.007
Directional asymmetry (R^2^)	0.0175	0.0115	-0.0094 ↔ 0.0159	-0.0004 ↔ 0.0159	0.069
Fluctuating asymmetry (MS)	0.0048	0.0026	0.00026 ↔ 0.00348	0.00137 ↔ 0.00348	0.001
Fluctuating asymmetry (R^2^)	0.1691	0.1386	-0.0410 ↔ 0.1003	-0.0019 ↔ 0.1003	0.058

BS = bootstrap; CI = confidence interval; MS = mean squares; R^2^ = coefficient of determination.

## Discussion

### Treatment of semilandmarks

Sliding of semilandmarks, either by minimum BE or minimum PD criteria, was shown to remove the effect of their initial arbitrary spacing along the analyzed outlines [37, 39]. The sliding step, contributiong to smoother transformations of one curve to another or to decrease in their Procrustes distances, may remove the supposedly artificial variation in the morphospace caused by the initial equidistant location of the semilandmarks [39]. In addition, sliding may increase discrimination among groups within the shape space [38]. On the other hand, it has been argued that sliding semilandmarks along tangential lines changes the actual morphology of the analyzed objects, which may introduce an artificial signal into the data [40]. It was also shown that semilandmark sliding in a dataset with relatively small variation may only slightly influence the actual shape representation of the objects [42]. However, increasing shape variation is supposed to enhance the importance of the sliding step regarding the final position of the points and, at the same time, two different sliding strategies may yield more disparate results [39]. In particular, the minimum PD criterion should be more susceptible to distortions in highly variable datasets [39] and this was clearly confirmed by our analyses. The shapes of *H*. *tuna* segments, analyzed in this study, were profoundly variable. For example, mean tangent PDs among the objects were about four to six times larger than that in the model dataset of Perez et al. [38]. This variation in outline shapes was clearly too large for a sensitive semilandmark sliding according to the minimum PD criterion. Indeed, individual semilandmarks were frequently slid behind their neighbors, which produced final configurations that visibly distorted the original morphology of the segments. Conversely, the minimum BE criterion and the superimposition of unslid equidistant points produced closely similar results. However, sliding still increased total variation in the superimposed data, including the asymmetric components. The minimum BE sliding step typically provides different densities of the semilandmarks in different parts of the outline. Thus, once this step is introduced, the differential density of the semilandmarks in some regions of the analyzed outline inevitably lead to increased shape difference between its original and mirrored configurations, thereby increasing the asymmetry of the dataset. Obviously, this does not happen when semilandmarks are kept in their equidistant positions, thereby resulting in a slightly lower shape variation.

### Morphological allometry of segment variation

Our analyses showed a conspicuous allometry in the shape of *H*. *tuna* segments. As the analyzed datasets did not include any non-calcified, immature segments, the observed allometric variation has to be ascribed to static allometry, which is based on the comparison of specimens at the same ontogenetic stage [56]; in this case the stage on mature, non-growing segments. Thus, the shape of smaller mature segments was consistently different from that of relatively larger segments. Mediterranean *H*. *tuna* is characterized by its bean-shaped or broadly oval segments [1, 57]. According to our analyses, these shapes correspond to the larger segments in both populations. Conversely, smaller segments fit the description of the deviant segments illustrated for several *Halimeda* species, including *H*. *tuna* [16]. These segments deviate from the typical morphology of individual species and may seriously hamper their morphometric identification. Interestingly, it has been shown that deviant segments preferably occur in the basal and apical parts of the branches, and apical deviant segments also included non-calcified, still growing segments [16]. Such segments were intentionally omitted in the present study to avoid confusing ontogenetic and static allometry. However, the static allometry observed in *H*. *tuna* can be explained by developmental heterochrony, which leads to a early termination of segment development in plants typical by relatively smaller fully calcified segments. This concurs with the observation that the shapes of the smaller mature segments reconstructed by our multivariate regression models were generally similar to those of the non-calcified segments reported by Verbruggen et al. [16].

The nested structure of our datasets, reflected by the multivariate Procrustes ANOVA models, actually means that the symmetric variation among segments was decomposed into several levels. The highest of these levels, the difference among plants from two different habitats, was highly related to allometry. In fact, because it was totally removed by prior accounting for size differences among segments, the variation in symmetrized segment shapes between populations was completely derived from their static allometry. Varying irradiation among habitats has been identified as one of the factors leading to size variation in *Halimeda* segments and thalli [17, 19, 21, 58]. This might have been reflected in our results, as *H*. *tuna* segments were significantly smaller in plants growing in the habitat typical by higher OSP values. Alternatively, the larger segments found in the population B might also be related to higher local nutrient loads in the habitat occupied by this population in relation to that occupied by the other studied population [17, 20]. However, published data for 2015 nitrate and phosphorus concentrations at the monitoring stations along the Slovenian coast do not support this assumption [59]. The locality A (Bay of St. Jernej) had about the same seasonal concentrations of phosphorus as the stations located close to the locality B at Fiesa, but the nitrate (NO_3_^-^-N) concentrations were about 40% lower than at the locality in Bay of St. Jernej, indicating that *H*. *tuna* populations at the locality B probably were not exposed to higher nutrient loads during the vegetative season preceding the sampling.

### Shape asymmetry of the segments

Besides symmetric variation, significant DA and FA in segment shapes within the plants were also detected. Subtle DA, which can be identified by geometric morphometrics, is typical for the bilaterally symmetric structures of most animals [15] and vascular plants [4, 60]. In mobile organisms, the developmental origins of DA have been related to genetically fixed developmental processes. Conversely, in plants, as well as in other sessile organisms, shape DA in multiple repeated parts of a single modular body can be caused by environmental heterogeneity that leads to differential growth of their symmetric parts [3, 7]. In *Halimeda* segments, the observed DA might be caused by the differences in irradiation of left and right halves of the segments forming a single branch that could be caused by partial shading of the overlaping branches due to complex branching patterns of *Halimeda* plants. This hypothesis should be tested using partially shaded thalli cultured under unilateral irradiation.

However, DA can often be dynamically interrelated with FA in bilaterally symmetric structures across individuals [61, 62], suggesting there might be a continuum of different components of asymmetry jointly responding to varying environmental factors [63]. This might be even more evident in the multiple repeated structures forming a single organism, such as *Halimeda* segments. Indeed, both DA and FA were elevated in the population A, which typically comprised plants with smaller segments than the population B. Total asymmetry, composed by both these components, was also inversely related to centroid size, which indicated an allometric relationship leading to more asymmetric shapes in relatively small segments. However, this allometry in the amount of shape asymmetry did not mask the differences in asymmetric variation between populations. Plants from Bay of St. Jernej had higher asymmetric loads than those from population B even in the analyses that accounted for the effect of size prior to evaluating the remaining effects. Several recent studies showed that shape FA is a very sensitive indicator of increased developmental stress in different benthic marine organisms, leading to high developmental instability, which mirrors suboptimal conditions for their morphogenesis due to anthropogenic pollution or other environmental factors [34–36]. Therefore, significant differences in shape asymmetry within plants from two studied populations might point out to possible environmentally related variation, which could not have been explained solely by morphological allometry.

Irradiation is inherently a continuous factor and the design of this study, consisting of plants sampled at just two discrete habitats, does not allow for a rigorous evaluation of its effects on the asymmetric components of the shape variation. However, we believe that our framework can be used as a basis for a research program involving formal evaluating of the relationship between FA, measured as a proxy of developmental instability at the plant level, and irradiation levels spanning a depth gradient throughout the sublittoral habitat.

Estimation of FA and DA values at the level of individual plants was based on two measures (MS and R^2^) yielded by the Procrustes ANOVA models. The MS values reflect the total amount of the variation in individual components of symmetry and asymmetry. These values were positively correlated at the level of individual *H*. *tuna* plants. Thus, plants with high shape variation among segments also often had high shape asymmetry between both segment halves. While morphogenesis of the segments composing the plants is a diachronic process, two halves of a single segment develop simultaneously. Therefore, it seems that if the morphogenetic precision of the segments actually reflects individual fittness of the plants, it can be evaluated both by shape variation among the segments and their bilateral asymmetry.

It should be noted that *H*. *tuna* plants inhabiting the northern-most area of the species distribution in the Northern Adriatic are relatively very small and composed by fewer segments than their counterparts from the central Mediterranean or tropical regions [1, 57, 64]. Therefore, should more asymmetric segments be expected in Northern Adriatic plants than in *H*. *tuna* from the core regions of the distribution area? Increased FA and total asymmetry were reported in marginal populations of several animal model systems [62, 65] and may reflect borderline environmental conditions at the margins of the distribution area. This hypothesis could be tested using a dataset covering a wider geographic and habitat range of the species.

Although nothing is known about the relationship between shape variation of the segments, including their asymmetry, and the calcification rates, this issue might be of much interest for future morphometric research of *Halimeda* populations. Calcification rates in *Halimeda* are decreased under lower light intensities [66]. Thus, less effective calcification might prolong the period of segment morphogenesis leading to their larger sizes and different shapes, which could then be ascribed to heterochrony among populations growing in habitats with different irradiation levels. In addition, it was also shown that the amount of CaCO_3_ in mature segments varies in relation to environmental factors, such as temperature or pH [67, 68]. Thus, morphometric variation of segments might then be studied in relation to calcification dynamics across environmental gradients in both recent and fossil *Halimeda* populations.

## Supporting information

S1 Dataset The x/y coordinates of the equidistant semilandmarks depicting the outlines of the 1964 segments as obtained from two separate digitizations. (TXT)

S1 Table Formulas used for obtaining the degrees of freedom, mean squares, and F-ratios in the nested Procrustes ANOVA models decomposing shape variation of the segments into different sources. (DOC)

S2 Table Results of the Procrustes ANOVA models evaluating the effect of each semilandmark treatment on the shape of the segments. (DOC)

S3 Table Summary of the PCA and the multivariate regression analyses. (DOC)

S4 Table Multivariate Procrustes ANOVA models based on the configurations obtained after sliding semilandmarks based on the minimum BE criterion, which decomposed symmetric variation and the components of asymmetry at the level of individual plants. (DOC)

S5 Table Linear correlation analyses among the MS obtained for the individual components of symmetry and asymmetry at the level of individual plants. (DOC)

S6 Table Multivariate Procrustes ANOVA models based on the configurations obtained from unslid equidistant semilandmarks, which decomposed symmetric variation and the components of asymmetry at the level of individual plants. (DOC)
