# Supplementary material for: Morphological allometry constrains symmetric shape variation, but not asymmetry, of Halimeda tuna (Bryopsidales, Ulvophyceae) segments
Source: PLoS One. 2018 Oct 25;13(10):e0206492. doi: 10.1371/journal.pone.0206492 (PMC6201959; doi:10.1371/journal.pone.0206492)
Supplement: S1 Table — (DOC) [file pone.0206492.s002.doc]

**S1 Table. Formulas used for obtaining the degrees of freedom, mean squares, and F-ratios in the nested Procrustes ANOVA models decomposing shape variation of the segments into different sources.**

| **Source of variation** | **abbreviation** | **df** | **MS** | **F** |
| --- | --- | --- | --- | --- |
| Locality | loc | n-1 | SS_ind_/n-1 | MS_loc_/MS_plt_ |
| Plant (locality) | plt | p-(n-1)-1 | SS_plt(loc)_/p-(n-1)-1 | MS_plt_/MS_sgm_ |
| Segment (plant) | sgm | s-[p-(n-1)-1]-1 | SS_sgm(plt)_/s-[p-(n-1)-1]-1 | MS_sgm_/MS_sgs_ |
| Side (plant) | sid | (d-1) × p | SS_sid(plt)_/[(d-1) × p] | MS_sid_/MS_sgs_ |
| Segment×side (plant) | sgs | s-[p-(n-1)-1]-1 | SS_sgs(plt)_/s-[p-(n-1)-1]-1 | MS_sgs_/MS_mre_ |
| Measurement error | mre | g × p | SS_mre_/(g × p) |  |

n = number of localities; p = number of plants; s = number of segments; d = number of symmetric parts per segment; g = number of digitisations.
