# Supplementary material for: Morphological allometry constrains symmetric shape variation, but not asymmetry, of Halimeda tuna (Bryopsidales, Ulvophyceae) segments
Source: PLoS One. 2018 Oct 25;13(10):e0206492. doi: 10.1371/journal.pone.0206492 (PMC6201959; doi:10.1371/journal.pone.0206492)
Supplement: S2 Table — (DOC) [file pone.0206492.s003.doc]

**S2 Table. Results of the Procrustes ANOVA models evaluating the effect of each semilandmark treatment on the shape of the segments.**

| **Unslid semilandmarks + minimum PD criterion** | | | | | |
| --- | --- | --- | --- | --- | --- |
| **Source of variation** | **df** | **SS** | **MS** | **F** | **p** |
| Segment | 981 | 44.740 | 0.0456 | 79.113 | 0.001 |
| **Segment×superimposition** | **982** | **6.630** | **0.0068** | **4.058** | **0.001** |
| Segment×digitisation | 982 | 1.634 | 0.0017 | 2.886 | 0.001 |
| Residuals | 982 | 0.566 | 0.0006 |  |  |
| Total | 3927 | 53.569 |  |  |  |
| **Unslid semilandmarks + minimum BE criterion** | | | | | |
| **Source of variation** | **df** | **SS** | **MS** | **F** | **p** |
| Segment | 981 | 67.220 | 0.0685 | 493.943 | 0.001 |
| **Segment×superimposition** | **982** | **1.799** | **0.0018** | **1.023** | **0.312** |
| Segment×digitisation | 982 | 1.758 | 0.0018 | 12.908 | 0.001 |
| Residuals | 982 | 0.136 | 0.0001 |  |  |
| Total | 3927 | 70.914 |  |  |  |
| **Minimum BE criterion + minimum PD criterion** | | | | | |
| **Source of variation** | **df** | **SS** | **MS** | **F** | **p** |
| Segment | 981 | 48.709 | 0.0497 | 59.131 | 0.001 |
| **Segment×superimposition** | **982** | **10.715** | **0.0109** | **5.819** | **0.001** |
| Segment**×**digitisation | 982 | 1.841 | 0.0019 | 2.233 | 0.001 |
| Residuals | 982 | 0.825 | 0.0008 |  |  |
| Total | 3927 | 62.089 |  |  |  |

BE = bending energy; PD = Procrustes distance; df = degrees of freedom; SS = sums of squares; MS = mean squares.

The **bold** lines in the models depict the effect of semilandmark treatment in individual segments tested against the effect of digitisation error on their shape.
