# Supplementary material for: Morphological allometry constrains symmetric shape variation, but not asymmetry, of Halimeda tuna (Bryopsidales, Ulvophyceae) segments
Source: PLoS One. 2018 Oct 25;13(10):e0206492. doi: 10.1371/journal.pone.0206492 (PMC6201959; doi:10.1371/journal.pone.0206492)
Supplement: S3 Table — (DOC) [file pone.0206492.s004.doc]

**S3 Table. Summary of the PCA and the multivariate regression analyses.**

| **GPA with unslid equidistant semilandmark** | | | | |
| --- | --- | --- | --- | --- |
| **Multivariate regression of shape on centroid size** | **Wilks' λ** | **p-value** | **% of the variation explained by the regression model** | **Fs, df1, df2** |
|  | 0.41 | 0.001 | 30.6 | 14.8, 176, 1787 |
| **PCA**  **(original and reflected / re-labelled data)** | **axis** | **singular value** | **% of the variation explained** | **symmetric / asymmetric variation** |
| Symmetric axes: 83.2% | PC1 | 6.35 | 67.7 | S |
| Asymmetric axes: 16.8% | PC2 | 2.42 | 9.9 | A |
|  | PC3 | 2.28 | 8.7 | S |
|  | PC4 | 1.40 | 3.3 | A |
|  | | | | |
| **GPA with semilandmarks slid according to the minimum BE criterion** | | | | |
| **Multivariate regression of shape on centroid size** | **Wilks' λ** | **p-value** | **% of the variation explained by the regression model** | **Fs, df1, df2** |
|  | 0.40 | 0.001 | 29.1 | 15.5, 176, 1787 |
| **PCA**  **(original and reflected / re-labelled data)** | **axis** | **singular value** | **% of the variation explained** | **symmetric / asymmetric variation** |
| Symmetric axes: 82.5% | PC1 | 6.99 | 65.2 | S |
| Asymmetric axes: 17.5% | PC2 | 2.74 | 10.0 | A |
|  | PC3 | 2.61 | 9.1 | S |
|  | PC4 | 1.62 | 3.5 | A |
