# Supplementary material for: Morphological allometry constrains symmetric shape variation, but not asymmetry, of Halimeda tuna (Bryopsidales, Ulvophyceae) segments
Source: PLoS One. 2018 Oct 25;13(10):e0206492. doi: 10.1371/journal.pone.0206492 (PMC6201959; doi:10.1371/journal.pone.0206492)
Supplement: S4 Table — (DOC) [file pone.0206492.s005.doc]

**S4 Table. Multivariate Procrustes ANOVA models based on the configurations obtained after sliding semilandmarks bsed on the minimum BE criterion, which decomposed symmetric variation and the components of asymmetry at the level of individual plants.**

| **Locality A, plant 01** | | | | | |
| --- | --- | --- | --- | --- | --- |
| **Source of variation** | **df** | **SS** | **MS** | **R^2^** | **F** |
| Segment | 14 | 0.6353 | 0.0454 | 0.735 | 3.393 |
| Side | 1 | 0.0195 | 0.0195 | 0.023 | 1.327 |
| Segment×Side | 14 | 0.1835 | 0.0131 | 0.212 | 10.445 |
| Measurement error | 30 | 0.0257 | 0.0009 | 0.030 |  |
| **Locality A, plant 02** | | | | | |
| **Source of variation** | **df** | **SS** | **MS** | **R^2^** | **F** |
| Segment | 5 | 0.1714 | 0.0343 | 0.825 | 6.179 |
| Side | 1 | 0.0029 | 0.0029 | 0.014 | 0.514 |
| Segment×Side | 5 | 0.0277 | 0.0055 | 0.134 | 11.739 |
| Measurement error | 12 | 0.0057 | 0.0005 | 0.027 |  |
| **Locality A, plant 03** | | | | | |
| **Source of variation** | **df** | **SS** | **MS** | **R^2^** | **F** |
| Segment | 5 | 0.2259 | 0.0452 | 0.567 | 3.251 |
| Side | 1 | 0.0897 | 0.0897 | 0.225 | 6.453 |
| Segment×Side | 5 | 0.0695 | 0.0139 | 0.174 | 12.382 |
| Measurement error | 12 | 0.0135 | 0.0011 | 0.034 |  |
| **Locality A, plant 04** | | | | | |
| **Source of variation** | **df** | **SS** | **MS** | **R^2^** | **F** |
| Segment | 11 | 0.2243 | 0.0204 | 0.534 | 1.458 |
| Side | 1 | 0.0150 | 0.0150 | 0.036 | 1.073 |
| Segment×Side | 11 | 0.1539 | 0.0139 | 0.366 | 12.401 |
| Measurement error | 24 | 0.0271 | 0.0011 | 0.064 |  |
| **Locality A, plant 05** | | | | | |
| **Source of variation** | **df** | **SS** | **MS** | **R^2^** | **F** |
| Segment | 7 | 0.1612 | 0.0230 | 0.829 | 8.879 |
| Side | 1 | 0.0055 | 0.0055 | 0.029 | 2.134 |
| Segment×Side | 7 | 0.0182 | 0.0026 | 0.093 | 4.408 |
| Measurement error | 16 | 0.0094 | 0.0006 | 0.049 |  |
| **Locality A, plant 06** | | | | | |
| **Source of variation** | **df** | **SS** | **MS** | **R^2^** | **F** |
| Segment | 9 | 0.5578 | 0.0619 | 0.872 | 10.154 |
| Side | 1 | 0.0116 | 0.0116 | 0.018 | 1.894 |
| Segment×Side | 9 | 0.0549 | 0.0061 | 0.086 | 7.837 |
| Measurement error | 20 | 0.0156 | 0.0008 | 0.024 |  |
| **Locality A, plant 07** | | | | | |
| **Source of variation** | **df** | **SS** | **MS** | **R^2^** | **F** |
| Segment | 7 | 0.1954 | 0.0279 | 0.853 | 9.301 |
| Side | 1 | 0.0038 | 0.0038 | 0.017 | 1.266 |
| Segment×Side | 7 | 0.0210 | 0.0030 | 0.092 | 5.472 |
| Measurement error | 16 | 0.0088 | 0.0005 | 0.038 |  |
| **Locality A, plant 08** | | | | | |
| **Source of variation** | **df** | **SS** | **MS** | **R^2^** | **F** |
| Segment | 6 | 0.5028 | 0.0838 | 0.702 | 3.488 |
| Side | 1 | 0.0479 | 0.0479 | 0.067 | 1.998 |
| Segment×Side | 6 | 0.1441 | 0.0240 | 0.201 | 16.036 |
| Measurement error | 14 | 0.0209 | 0.0015 | 0.029 |  |
| **Locality A, plant 09** | | | | | |
| **Source of variation** | **df** | **SS** | **MS** | **R^2^** | **F** |
| Segment | 6 | 0.1892 | 0.0315 | 0.864 | 9.949 |
| Side | 1 | 0.0059 | 0.0059 | 0.027 | 1.886 |
| Segment×Side | 6 | 0.0190 | 0.0032 | 0.087 | 9.434 |
| Measurement error | 14 | 0.0047 | 0.0003 | 0.022 |  |
| **Locality A, plant 10** | | | | | |
| **Source of variation** | **df** | **SS** | **MS** | **R^2^** | **F** |
| Segment | 9 | 0.2807 | 0.0312 | 0.782 | 4.458 |
| Side | 1 | 0.0099 | 0.0099 | 0.028 | 1.424 |
| Segment×Side | 9 | 0.0629 | 0.0069 | 0.175 | 25.475 |
| Measurement error | 20 | 0.0055 | 0.0003 | 0.015 |  |
| **Locality A, plant 11** | | | | | |
| **Source of variation** | **df** | **SS** | **MS** | **R^2^** | **F** |
| Segment | 11 | 0.1723 | 0.0157 | 0.718 | 3.323 |
| Side | 1 | 0.0038 | 0.0038 | 0.016 | 0.799 |
| Segment×Side | 11 | 0.0518 | 0.0047 | 0.216 | 9.342 |
| Measurement error | 24 | 0.0121 | 0.0005 | 0.050 |  |
| **Locality A, plant 12** | | | | | |
| **Source of variation** | **df** | **SS** | **MS** | **R^2^** | **F** |
| Segment | 8 | 0.2431 | 0.0304 | 0.770 | 4.407 |
| Side | 1 | 0.0076 | 0.0076 | 0.024 | 1.109 |
| Segment×Side | 8 | 0.0552 | 0.0069 | 0.175 | 12.819 |
| Measurement error | 18 | 0.0097 | 0.0005 | 0.031 |  |
| **Locality A, plant 13** | | | | | |
| **Source of variation** | **df** | **SS** | **MS** | **R^2^** | **F** |
| Segment | 14 | 0.4742 | 0.0339 | 0.845 | 6.553 |
| Side | 1 | 0.0043 | 0.0043 | 0.008 | 0.827 |
| Segment×Side | 14 | 0.0724 | 0.0052 | 0.129 | 15.032 |
| Measurement error | 30 | 0.0103 | 0.0003 | 0.018 |  |
| **Locality A, plant 14** | | | | | |
| **Source of variation** | **df** | **SS** | **MS** | **R^2^** | **F** |
| Segment | 9 | 0.2555 | 0.0284 | 0.734 | 5.235 |
| Side | 1 | 0.0142 | 0.0142 | 0.041 | 2.621 |
| Segment×Side | 9 | 0.0488 | 0.0054 | 0.140 | 3.647 |
| Measurement error | 20 | 0.0297 | 0.0015 | 0.085 |  |
| **Locality A, plant 15** | | | | | |
| **Source of variation** | **df** | **SS** | **MS** | **R^2^** | **F** |
| Segment | 10 | 0.251914 | 0.0252 | 0.773 | 5.180 |
| Side | 1 | 0.007738 | 0.0077 | 0.024 | 1.591 |
| Segment×Side | 10 | 0.048630 | 0.0049 | 0.149 | 6.019 |
| Measurement error | 22 | 0.017775 | 0.0008 | 0.055 |  |
| **Locality A, plant 16** | | | | | |
| **Source of variation** | **df** | **SS** | **MS** | **R^2^** | **F** |
| Segment | 8 | 0.4655 | 0.0582 | 0.805 | 5.191 |
| Side | 1 | 0.0074 | 0.0074 | 0.013 | 0.657 |
| Segment×Side | 8 | 0.0897 | 0.0112 | 0.155 | 12.757 |
| Measurement error | 18 | 0.0158 | 0.0009 | 0.027 |  |
| **Locality A, plant 17** | | | | | |
| **Source of variation** | **df** | **SS** | **MS** | **R^2^** | **F** |
| Segment | 6 | 0.2051 | 0.0342 | 0.811 | 6.432 |
| Side | 1 | 0.0083 | 0.0083 | 0.033 | 1.571 |
| Segment×Side | 6 | 0.0319 | 0.0053 | 0.126 | 9.756 |
| Measurement error | 14 | 0.0076 | 0.0005 | 0.030 |  |
| **Locality A, plant 18** | | | | | |
| **Source of variation** | **df** | **SS** | **MS** | **R^2^** | **F** |
| Segment | 15 | 0.2596 | 0.0173 | 0.627 | 2.085 |
| Side | 1 | 0.0103 | 0.0103 | 0.025 | 1.245 |
| Segment×Side | 15 | 0.1245 | 0.0083 | 0.301 | 13.643 |
| Measurement error | 32 | 0.0195 | 0.0006 | 0.047 |  |
| **Locality A, plant 19** | | | | | |
| **Source of variation** | **df** | **SS** | **MS** | **R^2^** | **F** |
| Segment | 8 | 0.2811 | 0.0351 | 0.856 | 7.401 |
| Side | 1 | 0.0036 | 0.0036 | 0.011 | 0.758 |
| Segment×Side | 8 | 0.0379 | 0.0047 | 0.116 | 15.094 |
| Measurement error | 18 | 0.0057 | 0.0003 | 0.017 |  |
| **Locality A, plant 20** | | | | | |
| **Source of variation** | **df** | **SS** | **MS** | **R^2^** | **F** |
| Segment | 10 | 0.2657 | 0.0266 | 0.751 | 3.932 |
| Side | 1 | 0.0071 | 0.0071 | 0.020 | 1.054 |
| Segment×Side | 10 | 0.0676 | 0.0068 | 0.191 | 11.139 |
| Measurement error | 22 | 0.0133 | 0.0006 | 0.038 |  |
| **Locality A, plant 21** | | | | | |
| **Source of variation** | **df** | **SS** | **MS** | **R^2^** | **F** |
| Segment | 7 | 0.2323 | 0.0332 | 0.784 | 4.802 |
| Side | 1 | 0.0028 | 0.0028 | 0.009 | 0.404 |
| Segment×Side | 7 | 0.0484 | 0.0069 | 0.163 | 8.499 |
| Measurement error | 16 | 0.0130 | 0.0008 | 0.044 |  |
| **Locality A, plant 22** | | | | | |
| **Source of variation** | **df** | **SS** | **MS** | **R^2^** | **F** |
| Segment | 8 | 0.2578 | 0.0322 | 0.752 | 4.841 |
| Side | 1 | 0.0169 | 0.0169 | 0.049 | 2.549 |
| Segment×Side | 8 | 0.0532 | 0.0067 | 0.155 | 8.146 |
| Measurement error | 18 | 0.0147 | 0.0008 | 0.043 |  |
| **Locality A, plant 23** | | | | | |
| **Source of variation** | **df** | **SS** | **MS** | **R^2^** | **F** |
| Segment | 9 | 0.4603 | 0.0511 | 0.784 | 4.710 |
| Side | 1 | 0.0136 | 0.0136 | 0.023 | 1.253 |
| Segment×Side | 9 | 0.0977 | 0.0109 | 0.166 | 13.989 |
| Measurement error | 20 | 0.0155 | 0.0008 | 0.026 |  |
| **Locality A, plant 24** | | | | | |
| **Source of variation** | **df** | **SS** | **MS** | **R^2^** | **F** |
| Segment | 9 | 0.1887 | 0.0209 | 0.723 | 3.538 |
| Side | 1 | 0.0039 | 0.0039 | 0.015 | 0.652 |
| Segment×Side | 9 | 0.0533 | 0.0059 | 0.204 | 7.824 |
| Measurement error | 20 | 0.0152 | 0.0008 | 0.058 |  |
| **Locality A, plant 25** | | | | | |
| **Source of variation** | **df** | **SS** | **MS** | **R^2^** | **F** |
| Segment | 10 | 0.3504 | 0.0350 | 0.609 | 2.155 |
| Side | 1 | 0.0378 | 0.0377 | 0.066 | 2.321 |
| Segment×Side | 10 | 0.1626 | 0.0163 | 0.282 | 14.349 |
| Measurement error | 22 | 0.0249 | 0.0011 | 0.043 |  |
| **Locality A, plant 26** | | | | | |
| **Source of variation** | **df** | **SS** | **MS** | **R^2^** | **F** |
| Segment | 8 | 0.4377 | 0.0547 | 0.859 | 7.463 |
| Side | 1 | 0.0047 | 0.0047 | 0.009 | 0.646 |
| Segment×Side | 8 | 0.0587 | 0.0073 | 0.115 | 16.025 |
| Measurement error | 18 | 0.0082 | 0.0005 | 0.016 |  |
| **Locality A, plant 27** | | | | | |
| **Source of variation** | **df** | **SS** | **MS** | **R^2^** | **F** |
| Segment | 7 | 0.3747 | 0.0535 | 0.782 | 6.590 |
| Side | 1 | 0.0238 | 0.0238 | 0.049 | 2.935 |
| Segment×Side | 7 | 0.0569 | 0.0081 | 0.119 | 5.444 |
| Measurement error | 16 | 0.0239 | 0.0015 | 0.049 |  |
| **Locality A, plant 28** | | | | | |
| **Source of variation** | **df** | **SS** | **MS** | **R^2^** | **F** |
| Segment | 9 | 0.288466 | 0.0321 | 0.754 | 4.137 |
| Side | 1 | 0.009197 | 0.0092 | 0.024 | 1.187 |
| Segment×Side | 9 | 0.069732 | 0.0077 | 0.182 | 10.235 |
| Measurement error | 20 | 0.015140 | 0.0008 | 0.039 |  |
| **Locality A, plant 29** | | | | | |
| **Source of variation** | **df** | **SS** | **MS** | **R^2^** | **F** |
| Segment | 9 | 0.0902 | 0.0100 | 0.631 | 2.331 |
| Side | 1 | 0.0029 | 0.0029 | 0.021 | 0.688 |
| Segment×Side | 9 | 0.0387 | 0.0043 | 0.271 | 7.819 |
| Measurement error | 20 | 0.0110 | 0.0006 | 0.077 |  |
| **Locality A, plant 30** | | | | | |
| **Source of variation** | **df** | **SS** | **MS** | **R^2^** | **F** |
| Segment | 11 | 0.3994 | 0.0363 | 0.895 | 13.329 |
| Side | 1 | 0.0057 | 0.0057 | 0.013 | 2.095 |
| Segment×Side | 11 | 0.0299 | 0.0027 | 0.067 | 5.740 |
| Measurement error | 24 | 0.0114 | 0.0005 | 0.026 |  |
| **Locality A, plant 31** | | | | | |
| **Source of variation** | **df** | **SS** | **MS** | **R^2^** | **F** |
| Segment | 6 | 0.1592 | 0.0265 | 0.844 | 9.785 |
| Side | 1 | 0.0090 | 0.0090 | 0.048 | 3.331 |
| Segment×Side | 6 | 0.0163 | 0.0027 | 0.086 | 8.996 |
| Measurement error | 14 | 0.0042 | 0.0003 | 0.022 |  |
| **Locality A, plant 32** | | | | | |
| **Source of variation** | **df** | **SS** | **MS** | **R^2^** | **F** |
| Segment | 12 | 0.0997 | 0.0083 | 0.545 | 1.434 |
| Side | 1 | 0.0065 | 0.0065 | 0.035 | 1.121 |
| Segment×Side | 12 | 0.0696 | 0.0058 | 0.379 | 20.486 |
| Measurement error | 26 | 0.0074 | 0.0003 | 0.040 |  |
| **Locality A, plant 33** | | | | | |
| **Source of variation** | **df** | **SS** | **MS** | **R^2^** | **F** |
| Segment | 6 | 0.3913 | 0.0652 | 0.708 | 2.898 |
| Side | 1 | 0.0165 | 0.0165 | 0.029 | 0.733 |
| Segment×Side | 6 | 0.1350 | 0.0225 | 0.244 | 32.231 |
| Measurement error | 14 | 0.0098 | 0.0007 | 0.018 |  |
| **Locality A, plant 34** | | | | | |
| **Source of variation** | **df** | **SS** | **MS** | **R^2^** | **F** |
| Segment | 6 | 0.0524 | 0.0087 | 0.551 | 1.705 |
| Side | 1 | 0.0095 | 0.0095 | 0.099 | 1.852 |
| Segment×Side | 6 | 0.0307 | 0.0052 | 0.323 | 28.019 |
| Measurement error | 14 | 0.0026 | 0.0002 | 0.027 |  |
| **Locality A, plant 35** | | | | | |
| **Source of variation** | **df** | **SS** | **MS** | **R^2^** | **F** |
| Segment | 5 | 0.2941 | 0.0588 | 0.932 | 28.919 |
| Side | 1 | 0.0081 | 0.0081 | 0.026 | 3.991 |
| Segment×Side | 5 | 0.0102 | 0.0020 | 0.032 | 7.572 |
| Measurement error | 12 | 0.0032 | 0.0003 | 0.010 |  |
| **Locality A, plant 36** | | | | | |
| **Source of variation** | **df** | **SS** | **MS** | **R^2^** | **F** |
| Segment | 16 | 0.5203 | 0.0325 | 0.801 | 5.833 |
| Side | 1 | 0.0073 | 0.0073 | 0.011 | 1.309 |
| Segment×Side | 16 | 0.0892 | 0.0056 | 0.137 | 5.855 |
| Measurement error | 34 | 0.0324 | 0.0009 | 0.049 |  |
| **Locality A, plant 37** | | | | | |
| **Source of variation** | **df** | **SS** | **MS** | **R^2^** | **F** |
| Segment | 7 | 0.0746 | 0.0107 | 0.669 | 3.270 |
| Side | 1 | 0.0072 | 0.0072 | 0.064 | 2.198 |
| Segment×Side | 7 | 0.0228 | 0.0033 | 0.205 | 7.616 |
| Measurement error | 16 | 0.0068 | 0.0004 | 0.061 |  |
| **Locality A, plant 38** | | | | | |
| **Source of variation** | **df** | **SS** | **MS** | **R^2^** | **F** |
| Segment | 6 | 0.2975 | 0.0496 | 0.836 | 7.967 |
| Side | 1 | 0.0117 | 0.0117 | 0.033 | 1.873 |
| Segment×Side | 6 | 0.0373 | 0.0062 | 0.105 | 9.336 |
| Measurement error | 14 | 0.0093 | 0.0007 | 0.026 |  |
| **Locality A, plant 39** | | | | | |
| **Source of variation** | **df** | **SS** | **MS** | **R^2^** | **F** |
| Segment | 5 | 0.0909 | 0.0182 | 0.738 | 6.616 |
| Side | 1 | 0.0135 | 0.0135 | 0.109 | 4.896 |
| Segment×Side | 5 | 0.0138 | 0.0028 | 0.112 | 6.483 |
| Measurement error | 12 | 0.0051 | 0.0004 | 0.041 |  |
| **Locality A, plant 40** | | | | | |
| **Source of variation** | **df** | **SS** | **MS** | **R^2^** | **F** |
| Segment | 8 | 0.1884 | 0.0235 | 0.756 | 3.678 |
| Side | 1 | 0.0035 | 0.0035 | 0.014 | 0.545 |
| Segment×Side | 8 | 0.0512 | 0.0064 | 0.206 | 18.936 |
| Measurement error | 18 | 0.0061 | 0.0003 | 0.024 |  |
| **Locality A, plant 41** | | | | | |
| **Source of variation** | **df** | **SS** | **MS** | **R^2^** | **F** |
| Segment | 11 | 0.3168 | 0.0288 | 0.771 | 4.086 |
| Side | 1 | 0.0056 | 0.0056 | 0.014 | 0.793 |
| Segment×Side | 11 | 0.0775 | 0.0070 | 0.189 | 15.476 |
| Measurement error | 24 | 0.0109 | 0.0005 | 0.027 |  |
| **Locality A, plant 42** | | | | | |
| **Source of variation** | **df** | **SS** | **MS** | **R^2^** | **F** |
| Segment | 12 | 0.3581 | 0.0298 | 0.762 | 4.349 |
| Side | 1 | 0.0074 | 0.0074 | 0.016 | 1.072 |
| Segment×Side | 12 | 0.0823 | 0.0069 | 0.175 | 8.005 |
| Measurement error | 26 | 0.0223 | 0.0009 | 0.047 |  |
| **Locality A, plant 43** | | | | | |
| **Source of variation** | **df** | **SS** | **MS** | **R^2^** | **F** |
| Segment | 14 | 0.3886 | 0.0278 | 0.766 | 4.481 |
| Side | 1 | 0.0152 | 0.0152 | 0.029 | 2.457 |
| Segment×Side | 14 | 0.0867 | 0.0062 | 0.171 | 11.068 |
| Measurement error | 30 | 0.0168 | 0.0006 | 0.033 |  |
| **Locality A, plant 44** | | | | | |
| **Source of variation** | **df** | **SS** | **MS** | **R^2^** | **F** |
| Segment | 9 | 0.3007 | 0.0334 | 0.743 | 3.960 |
| Side | 1 | 0.0167 | 0.0167 | 0.041 | 1.975 |
| Segment×Side | 9 | 0.0759 | 0.0084 | 0.188 | 14.995 |
| Measurement error | 20 | 0.0113 | 0.0006 | 0.028 |  |
| **Locality A, plant 45** | | | | | |
| **Source of variation** | **df** | **SS** | **MS** | **R^2^** | **F** |
| Segment | 16 | 0.6897 | 0.0431 | 0.837 | 8.092 |
| Side | 1 | 0.0059 | 0.0059 | 0.007 | 1.107 |
| Segment×Side | 16 | 0.0852 | 0.0053 | 0.103 | 4.189 |
| Measurement error | 34 | 0.0432 | 0.0013 | 0.052 |  |
| **Locality A, plant 46** | | | | | |
| **Source of variation** | **df** | **SS** | **MS** | **R^2^** | **F** |
| Segment | 8 | 0.2993 | 0.0374 | 0.693 | 2.569 |
| Side | 1 | 0.0066 | 0.0066 | 0.015 | 0.456 |
| Segment×Side | 8 | 0.1165 | 0.0146 | 0.269 | 28.500 |
| Measurement error | 18 | 0.0092 | 0.0005 | 0.021 |  |
| **Locality A, plant 47** | | | | | |
| **Source of variation** | **df** | **SS** | **MS** | **R^2^** | **F** |
| Segment | 9 | 0.1802 | 0.0200 | 0.681 | 2.904 |
| Side | 1 | 0.0089 | 0.0089 | 0.033 | 1.285 |
| Segment×Side | 9 | 0.0620 | 0.0069 | 0.234 | 10.171 |
| Measurement error | 20 | 0.0136 | 0.0007 | 0.051 |  |
| **Locality A, plant 48** | | | | | |
| **Source of variation** | **df** | **SS** | **MS** | **R^2^** | **F** |
| Segment | 7 | 0.0962 | 0.0137 | 0.609 | 2.419 |
| Side | 1 | 0.0153 | 0.0153 | 0.097 | 2.690 |
| Segment×Side | 7 | 0.0398 | 0.0057 | 0.252 | 13.403 |
| Measurement error | 16 | 0.0068 | 0.0004 | 0.043 |  |
| **Locality B, plant 01** | | | | | |
| **Source of variation** | **df** | **SS** | **MS** | **R^2^** | **F** |
| Segment | 9 | 0.2593 | 0.0288 | 0.782 | 6.566 |
| Side | 1 | 0.0043 | 0.0043 | 0.013 | 0.972 |
| Segment×Side | 9 | 0.0395 | 0.0044 | 0.119 | 3.077 |
| Measurement error | 20 | 0.0285 | 0.0014 | 0.086 |  |
| **Locality B, plant 02** | | | | | |
| **Source of variation** | **df** | **SS** | **MS** | **R^2^** | **F** |
| Segment | 8 | 0.3606 | 0.0451 | 0.835 | 10.702 |
| Side | 1 | 0.0099 | 0.0099 | 0.023 | 2.344 |
| Segment×Side | 8 | 0.0337 | 0.0042 | 0.078 | 2.734 |
| Measurement error | 18 | 0.0277 | 0.0015 | 0.064 |  |
| **Locality B, plant 03** | | | | | |
| **Source of variation** | **df** | **SS** | **MS** | **R^2^** | **F** |
| Segment | 9 | 0.4916 | 0.0546 | 0.887 | 13.038 |
| Side | 1 | 0.0055 | 0.0055 | 0.009 | 1.309 |
| Segment×Side | 9 | 0.0377 | 0.0042 | 0.068 | 4.379 |
| Measurement error | 20 | 0.0191 | 0.0009 | 0.035 |  |
| **Locality B, plant 04** | | | | | |
| **Source of variation** | **df** | **SS** | **MS** | **R^2^** | **F** |
| Segment | 9 | 0.3732 | 0.0415 | 0.811 | 5.399 |
| Side | 1 | 0.0042 | 0.0042 | 0.009 | 0.546 |
| Segment×Side | 9 | 0.0691 | 0.0077 | 0.150 | 11.396 |
| Measurement error | 20 | 0.0135 | 0.0007 | 0.029 |  |
| **Locality B, plant 05** | | | | | |
| **Source of variation** | **df** | **SS** | **MS** | **R^2^** | **F** |
| Segment | 7 | 0.1783 | 0.0255 | 0.689 | 2.941 |
| Side | 1 | 0.0132 | 0.0132 | 0.051 | 1.519 |
| Segment×Side | 7 | 0.0606 | 0.0087 | 0.234 | 20.840 |
| Measurement error | 16 | 0.0067 | 0.0004 | 0.026 |  |
| **Locality B, plant 06** | | | | | |
| **Source of variation** | **df** | **SS** | **MS** | **R^2^** | **F** |
| Segment | 7 | 0.2344 | 0.0335 | 0.825 | 6.487 |
| Side | 1 | 0.0029 | 0.0029 | 0.010 | 0.556 |
| Segment×Side | 7 | 0.0361 | 0.0052 | 0.127 | 7.613 |
| Measurement error | 16 | 0.0109 | 0.0007 | 0.038 |  |
| **Locality B, plant 07** | | | | | |
| **Source of variation** | **df** | **SS** | **MS** | **R^2^** | **F** |
| Segment | 8 | 0.2003 | 0.0250 | 0.809 | 5.730 |
| Side | 1 | 0.0055 | 0.0055 | 0.022 | 1.248 |
| Segment×Side | 8 | 0.0349 | 0.0044 | 0.141 | 11.693 |
| Measurement error | 18 | 0.0067 | 0.0004 | 0.027 |  |
| **Locality B, plant 08** | | | | | |
| **Source of variation** | **df** | **SS** | **MS** | **R^2^** | **F** |
| Segment | 7 | 0.1530 | 0.0219 | 0.864 | 15.285 |
| Side | 1 | 0.0080 | 0.0080 | 0.045 | 5.622 |
| Segment×Side | 7 | 0.0100 | 0.0014 | 0.057 | 3.841 |
| Measurement error | 16 | 0.0059 | 0.0004 | 0.034 |  |
| **Locality B, plant 09** | | | | | |
| **Source of variation** | **df** | **SS** | **MS** | **R^2^** | **F** |
| Segment | 9 | 0.2052 | 0.0228 | 0.831 | 7.104 |
| Side | 1 | 0.0074 | 0.0074 | 0.029 | 2.290 |
| Segment×Side | 9 | 0.0289 | 0.0032 | 0.117 | 11.499 |
| Measurement error | 20 | 0.0056 | 0.0003 | 0.023 |  |
| **Locality B, plant 10** | | | | | |
| **Source of variation** | **df** | **SS** | **MS** | **R^2^** | **F** |
| Segment | 7 | 0.2701 | 0.0386 | 0.837 | 6.481 |
| Side | 1 | 0.0047 | 0.0047 | 0.015 | 0.796 |
| Segment×Side | 7 | 0.0417 | 0.0059 | 0.129 | 15.071 |
| Measurement error | 16 | 0.0063 | 0.0004 | 0.019 |  |
| **Locality B, plant 11** | | | | | |
| **Source of variation** | **df** | **SS** | **MS** | **R^2^** | **F** |
| Segment | 8 | 0.1636 | 0.0205 | 0.609 | 2.019 |
| Side | 1 | 0.0075 | 0.0075 | 0.028 | 0.742 |
| Segment×Side | 8 | 0.0810 | 0.0101 | 0.302 | 11.218 |
| Measurement error | 18 | 0.0162 | 0.0009 | 0.061 |  |
| **Locality B, plant 12** | | | | | |
| **Source of variation** | **df** | **SS** | **MS** | **R^2^** | **F** |
| Segment | 12 | 0.2022 | 0.0168 | 0.598 | 1.909 |
| Side | 1 | 0.0107 | 0.0107 | 0.032 | 1.210 |
| Segment×Side | 12 | 0.1059 | 0.0088 | 0.313 | 11.960 |
| Measurement error | 26 | 0.0192 | 0.0007 | 0.057 |  |
| **Locality B, plant 13** | | | | | |
| **Source of variation** | **df** | **SS** | **MS** | **R^2^** | **F** |
| Segment | 8 | 0.1058 | 0.0132 | 0.769 | 5.654 |
| Side | 1 | 0.0016 | 0.0016 | 0.012 | 0.689 |
| Segment×Side | 8 | 0.0187 | 0.0023 | 0.136 | 3.647 |
| Measurement error | 18 | 0.0115 | 0.0006 | 0.084 |  |
| **Locality B, plant 14** | | | | | |
| **Source of variation** | **df** | **SS** | **MS** | **R^2^** | **F** |
| Segment | 15 | 0.4652 | 0.0310 | 0.859 | 8.542 |
| Side | 1 | 0.0070 | 0.0070 | 0.013 | 1.939 |
| Segment×Side | 15 | 0.0545 | 0.0036 | 0.101 | 7.679 |
| Measurement error | 32 | 0.0151 | 0.0004 | 0.028 |  |
| **Locality B, plant 15** | | | | | |
| **Source of variation** | **df** | **SS** | **MS** | **R^2^** | **F** |
| Segment | 14 | 0.5606 | 0.0400 | 0.908 | 19.389 |
| Side | 1 | 0.0099 | 0.0099 | 0.016 | 4.798 |
| Segment×Side | 14 | 0.0289 | 0.0021 | 0.047 | 3.510 |
| Measurement error | 30 | 0.0177 | 0.0006 | 0.029 |  |
| **Locality B, plant 16** | | | | | |
| **Source of variation** | **df** | **SS** | **MS** | **R^2^** | **F** |
| Segment | 6 | 0.0937 | 0.0156 | 0.782 | 4.622 |
| Side | 1 | 0.0019 | 0.0019 | 0.017 | 0.589 |
| Segment×Side | 6 | 0.0203 | 0.0034 | 0.169 | 12.082 |
| Measurement error | 14 | 0.0039 | 0.0003 | 0.033 |  |
| **Locality B, plant 17** | | | | | |
| **Source of variation** | **df** | **SS** | **MS** | **R^2^** | **F** |
| Segment | 23 | 0.5554 | 0.0241 | 0.688 | 2.479 |
| Side | 1 | 0.0018 | 0.0018 | 0.002 | 0.185 |
| Segment×Side | 23 | 0.2241 | 0.0097 | 0.278 | 18.113 |
| Measurement error | 48 | 0.0258 | 0.0005 | 0.032 |  |
| **Locality B, plant 18** | | | | | |
| **Source of variation** | **df** | **SS** | **MS** | **R^2^** | **F** |
| Segment | 8 | 0.2629 | 0.0329 | 0.847 | 8.003 |
| Side | 1 | 0.0062 | 0.0062 | 0.019 | 1.506 |
| Segment×Side | 8 | 0.0329 | 0.0041 | 0.106 | 8.811 |
| Measurement error | 18 | 0.0084 | 0.0005 | 0.027 |  |
| **Locality B, plant 19** | | | | | |
| **Source of variation** | **df** | **SS** | **MS** | **R^2^** | **F** |
| Segment | 13 | 0.2934 | 0.0226 | 0.795 | 4.796 |
| Side | 1 | 0.0038 | 0.0038 | 0.010 | 0.812 |
| Segment×Side | 13 | 0.0612 | 0.0047 | 0.166 | 12.306 |
| Measurement error | 28 | 0.0107 | 0.0004 | 0.029 |  |
| **Locality B, plant 20** | | | | | |
| **Source of variation** | **df** | **SS** | **MS** | **R^2^** | **F** |
| Segment | 21 | 0.4075 | 0.0194 | 0.765 | 3.888 |
| Side | 1 | 0.0013 | 0.0013 | 0.002 | 0.262 |
| Segment×Side | 21 | 0.1048 | 0.0049 | 0.197 | 11.619 |
| Measurement error | 44 | 0.0189 | 0.0004 | 0.035 |  |
| **Locality B, plant 21** | | | | | |
| **Source of variation** | **df** | **SS** | **MS** | **R^2^** | **F** |
| Segment | 9 | 0.1185 | 0.0132 | 0.755 | 3.938 |
| Side | 1 | 0.0022 | 0.0022 | 0.014 | 0.661 |
| Segment×Side | 9 | 0.0301 | 0.0033 | 0.192 | 10.838 |
| Measurement error | 20 | 0.0062 | 0.0003 | 0.039 |  |
| **Locality B, plant 22** | | | | | |
| **Source of variation** | **df** | **SS** | **MS** | **R^2^** | **F** |
| Segment | 7 | 0.1005 | 0.0144 | 0.8827 | 10.656 |
| Side | 1 | 0.0017 | 0.0017 | 0.0146 | 1.234 |
| Segment×Side | 7 | 0.0094 | 0.0013 | 0.0828 | 9.541 |
| Measurement error | 16 | 0.0023 | 0.0001 | 0.0199 |  |
| **Locality B, plant 23** | | | | | |
| **Source of variation** | **df** | **SS** | **MS** | **R^2^** | **F** |
| Segment | 17 | 0.3187 | 0.0187 | 0.818 | 6.296 |
| Side | 1 | 0.0044 | 0.0044 | 0.011 | 1.473 |
| Segment×Side | 17 | 0.0506 | 0.0029 | 0.129 | 6.806 |
| Measurement error | 36 | 0.0158 | 0.0004 | 0.040 |  |
| **Locality B, plant 24** | | | | | |
| **Source of variation** | **df** | **SS** | **MS** | **R^2^** | **F** |
| Segment | 14 | 0.1713 | 0.0122 | 0.790 | 5.735 |
| Side | 1 | 0.0063 | 0.0063 | 0.029 | 2.967 |
| Segment×Side | 14 | 0.0299 | 0.0021 | 0.138 | 6.940 |
| Measurement error | 30 | 0.0092 | 0.0003 | 0.043 |  |
| **Locality B, plant 25** | | | | | |
| **Source of variation** | **df** | **SS** | **MS** | **R^2^** | **F** |
| Segment | 8 | 0.1939 | 0.0242 | 0.874 | 9.896 |
| Side | 1 | 0.0020 | 0.0020 | 0.009 | 0.818 |
| Segment×Side | 8 | 0.0196 | 0.0024 | 0.088 | 7.029 |
| Measurement error | 18 | 0.0063 | 0.0003 | 0.029 |  |
| **Locality B, plant 26** | | | | | |
| **Source of variation** | **df** | **SS** | **MS** | **R^2^** | **F** |
| Segment | 9 | 0.0324 | 0.0036 | 0.456 | 1.078 |
| Side | 1 | 0.0029 | 0.0029 | 0.040 | 0.860 |
| Segment×Side | 9 | 0.0301 | 0.0033 | 0.423 | 11.742 |
| Measurement error | 20 | 0.0057 | 0.0003 | 0.080 |  |
| **Locality B, plant 27** | | | | | |
| **Source of variation** | **df** | **SS** | **MS** | **R^2^** | **F** |
| Segment | 7 | 0.3288 | 0.0469 | 0.887 | 9.903 |
| Side | 1 | 0.0039 | 0.0039 | 0.010 | 0.816 |
| Segment×Side | 7 | 0.0332 | 0.0047 | 0.089 | 15.546 |
| Measurement error | 16 | 0.0049 | 0.0003 | 0.013 |  |
| **Locality B, plant 28** | | | | | |
| **Source of variation** | **df** | **SS** | **MS** | **R^2^** | **F** |
| Segment | 5 | 0.1681 | 0.0336 | 0.902 | 17.053 |
| Side | 1 | 0.0054 | 0.0054 | 0.029 | 2.738 |
| Segment×Side | 5 | 0.0099 | 0.0019 | 0.053 | 7.679 |
| Measurement error | 12 | 0.0031 | 0.0003 | 0.017 |  |
| **Locality B, plant 29** | | | | | |
| **Source of variation** | **df** | **SS** | **MS** | **R^2^** | **F** |
| Segment | 6 | 0.0351 | 0.0059 | 0.554 | 1.744 |
| Side | 1 | 0.0037 | 0.0037 | 0.059 | 1.113 |
| Segment×Side | 6 | 0.0201 | 0.0034 | 0.318 | 10.722 |
| Measurement error | 14 | 0.0044 | 0.0003 | 0.069 |  |
| **Locality B, plant 30** | | | | | |
| **Source of variation** | **df** | **SS** | **MS** | **R^2^** | **F** |
| Segment | 9 | 0.1235 | 0.0137 | 0.708 | 3.787 |
| Side | 1 | 0.0034 | 0.0034 | 0.019 | 0.939 |
| Segment×Side | 9 | 0.0326 | 0.0036 | 0.187 | 4.844 |
| Measurement error | 20 | 0.0149 | 0.0007 | 0.086 |  |
| **Locality B, plant 31** | | | | | |
| **Source of variation** | **df** | **SS** | **MS** | **R^2^** | **F** |
| Segment | 7 | 0.1929 | 0.0276 | 0.704 | 3.097 |
| Side | 1 | 0.0116 | 0.0116 | 0.042 | 1.300 |
| Segment×Side | 7 | 0.0623 | 0.0089 | 0.227 | 19.967 |
| Measurement error | 16 | 0.0071 | 0.0004 | 0.026 |  |
| **Locality B, plant 32** | | | | | |
| **Source of variation** | **df** | **SS** | **MS** | **R^2^** | **F** |
| Segment | 7 | 0.1657 | 0.0237 | 0.715 | 3.819 |
| Side | 1 | 0.0175 | 0.0175 | 0.076 | 2.825 |
| Segment×Side | 7 | 0.0434 | 0.0062 | 0.187 | 18.917 |
| Measurement error | 16 | 0.0052 | 0.0003 | 0.023 |  |
| **Locality B, plant 33** | | | | | |
| **Source of variation** | **df** | **SS** | **MS** | **R^2^** | **F** |
| Segment | 11 | 0.2523 | 0.0229 | 0.675 | 2.280 |
| Side | 1 | 0.0025 | 0.0025 | 0.007 | 0.245 |
| Segment×Side | 11 | 0.1106 | 0.0101 | 0.296 | 28.715 |
| Measurement error | 24 | 0.0084 | 0.0004 | 0.022 |  |
| **Locality B, plant 34** | | | | | |
| **Source of variation** | **df** | **SS** | **MS** | **R^2^** | **F** |
| Segment | 7 | 0.1629 | 0.0233 | 0.885 | 10.932 |
| Side | 1 | 0.0022 | 0.0022 | 0.012 | 1.016 |
| Segment×Side | 7 | 0.0149 | 0.0021 | 0.081 | 8.369 |
| Measurement error | 16 | 0.0041 | 0.0003 | 0.022 |  |
| **Locality B, plant 35** | | | | | |
| **Source of variation** | **df** | **SS** | **MS** | **R^2^** | **F** |
| Segment | 8 | 0.2123 | 0.0265 | 0.901 | 15.838 |
| Side | 1 | 0.0059 | 0.0059 | 0.025 | 3.557 |
| Segment×Side | 8 | 0.0134 | 0.0017 | 0.057 | 7.798 |
| Measurement error | 18 | 0.0039 | 0.0002 | 0.016 |  |
| **Locality B, plant 36** | | | | | |
| **Source of variation** | **df** | **SS** | **MS** | **R^2^** | **F** |
| Segment | 6 | 0.1821 | 0.0304 | 0.895 | 11.736 |
| Side | 1 | 0.0026 | 0.0026 | 0.013 | 0.990 |
| Segment×Side | 6 | 0.0155 | 0.0026 | 0.076 | 10.894 |
| Measurement error | 14 | 0.0033 | 0.0002 | 0.016 |  |
| **Locality B, plant 37** | | | | | |
| **Source of variation** | **df** | **SS** | **MS** | **R^2^** | **F** |
| Segment | 9 | 0.0581 | 0.0065 | 0.649 | 3.072 |
| Side | 1 | 0.0017 | 0.0017 | 0.019 | 0.812 |
| Segment×Side | 9 | 0.0189 | 0.0021 | 0.211 | 3.923 |
| Measurement error | 20 | 0.0107 | 0.0005 | 0.119 |  |
| **Locality B, plant 38** | | | | | |
| **Source of variation** | **df** | **SS** | **MS** | **R^2^** | **F** |
| Segment | 8 | 0.1662 | 0.0208 | 0.747 | 3.565 |
| Side | 1 | 0.0049 | 0.0049 | 0.022 | 0.848 |
| Segment×Side | 8 | 0.0466 | 0.0058 | 0.209 | 22.288 |
| Measurement error | 18 | 0.0047 | 0.0003 | 0.021 |  |
| **Locality B, plant 39** | | | | | |
| **Source of variation** | **df** | **SS** | **MS** | **R^2^** | **F** |
| Segment | 8 | 0.1044 | 0.0131 | 0.671 | 2.392 |
| Side | 1 | 0.0025 | 0.0025 | 0.016 | 0.465 |
| Segment×Side | 8 | 0.0437 | 0.0055 | 0.281 | 20.004 |
| Measurement error | 18 | 0.0049 | 0.0003 | 0.032 |  |
| **Locality B, plant 40** | | | | | |
| **Source of variation** | **df** | **SS** | **MS** | **R^2^** | **F** |
| Segment | 9 | 0.1074 | 0.0119 | 0.759 | 3.805 |
| Side | 1 | 0.0010 | 0.0010 | 0.007 | 0.334 |
| Segment×Side | 9 | 0.0282 | 0.0031 | 0.199 | 12.852 |
| Measurement error | 20 | 0.0049 | 0.0002 | 0.034 |  |
| **Locality B, plant 41** | | | | | |
| **Source of variation** | **df** | **SS** | **MS** | **R^2^** | **F** |
| Segment | 10 | 0.1445 | 0.0145 | 0.747 | 5.075 |
| Side | 1 | 0.0136 | 0.0136 | 0.070 | 4.769 |
| Segment×Side | 10 | 0.0285 | 0.0028 | 0.147 | 9.052 |
| Measurement error | 22 | 0.0069 | 0.0003 | 0.036 |  |
| **Locality B, plant 42** | | | | | |
| **Source of variation** | **df** | **SS** | **MS** | **R^2^** | **F** |
| Segment | 6 | 0.1636 | 0.0273 | 0.755 | 6.418 |
| Side | 1 | 0.0229 | 0.0229 | 0.106 | 5.392 |
| Segment×Side | 6 | 0.0255 | 0.0042 | 0.118 | 13.060 |
| Measurement error | 14 | 0.0046 | 0.0003 | 0.021 |  |
| **Locality B, plant 43** | | | | | |
| **Source of variation** | **df** | **SS** | **MS** | **R^2^** | **F** |
| Segment | 11 | 0.2849 | 0.0258 | 0.869 | 9.753 |
| Side | 1 | 0.0033 | 0.0033 | 0.010 | 1.244 |
| Segment×Side | 11 | 0.0292 | 0.0027 | 0.089 | 6.318 |
| Measurement error | 24 | 0.0101 | 0.0004 | 0.031 |  |
| **Locality B, plant 44** | | | | | |
| **Source of variation** | **df** | **SS** | **MS** | **R^2^** | **F** |
| Segment | 13 | 0.4373 | 0.0336 | 0.899 | 12.601 |
| Side | 1 | 0.0059 | 0.0059 | 0.012 | 2.228 |
| Segment×Side | 13 | 0.0347 | 0.0027 | 0.071 | 9.109 |
| Measurement error | 28 | 0.0082 | 0.0003 | 0.017 |  |
| **Locality B, plant 45** | | | | | |
| **Source of variation** | **df** | **SS** | **MS** | **R^2^** | **F** |
| Segment | 11 | 0.2078 | 0.0189 | 0.796 | 5.656 |
| Side | 1 | 0.0046 | 0.0046 | 0.018 | 1.373 |
| Segment×Side | 11 | 0.0367 | 0.0033 | 0.141 | 6.690 |
| Measurement error | 24 | 0.0119 | 0.0005 | 0.046 |  |
| **Locality B, plant 46** | | | | | |
| **Source of variation** | **df** | **SS** | **MS** | **R^2^** | **F** |
| Segment | 6 | 0.0509 | 0.0085 | 0.563 | 2.552 |
| Side | 1 | 0.0099 | 0.0099 | 0.109 | 2.963 |
| Segment×Side | 6 | 0.0199 | 0.0033 | 0.221 | 4.812 |
| Measurement error | 14 | 0.0097 | 0.0007 | 0.107 |  |
| **Locality B, plant 47** | | | | | |
| **Source of variation** | **df** | **SS** | **MS** | **R^2^** | **F** |
| Segment | 10 | 0.2251 | 0.0225 | 0.669 | 2.872 |
| Side | 1 | 0.0065 | 0.0065 | 0.019 | 0.824 |
| Segment×Side | 10 | 0.0784 | 0.0078 | 0.233 | 6.465 |
| Measurement error | 22 | 0.0267 | 0.0012 | 0.079 |  |
| **Locality B, plant 48** | | | | | |
| **Source of variation** | **df** | **SS** | **MS** | **R^2^** | **F** |
| Segment | 12 | 0.3616 | 0.0301 | 0.829 | 7.529 |
| Side | 1 | 0.0149 | 0.0149 | 0.034 | 3.729 |
| Segment×Side | 12 | 0.0480 | 0.0040 | 0.110 | 9.050 |
| Measurement error | 26 | 0.0115 | 0.0004 | 0.026 |  |

df = degrees of freedom; SS = sums of squares; MS = mean squares, R^2^ = coefficient of determination.
