# Supplementary material for: Morphological allometry constrains symmetric shape variation, but not asymmetry, of Halimeda tuna (Bryopsidales, Ulvophyceae) segments
Source: PLoS One. 2018 Oct 25;13(10):e0206492. doi: 10.1371/journal.pone.0206492 (PMC6201959; doi:10.1371/journal.pone.0206492)
Supplement: S5 Table — (DOC) [file pone.0206492.s006.doc]

**S5 Table. Linear correlation analyses among the MS obtained for the individual components of symmetry and asymmetry at the level of individual plants.**

| **Minimum BE criterion** | | | |
| --- | --- | --- | --- |
|  | Pearson's r | R^2^ | *p*-value |
| Symmetry vs. DA | 0.37 | 0.13 | 0.0013 |
| Symmetry vs. FA | 0.51 | 0.26 | 0.0001 |
| DA vs. FA | 0.57 | 0.32 | 0.0002 |
| **Unslid semilandmarks** | | | |
|  | Pearson's r | R^2^ | *p*-value |
| Symmetry vs. DA | 0.32 | 0.10 | 0.0061 |
| Symmetry vs. FA | 0.54 | 0.29 | 0.0001 |
| DA vs. FA | 0.46 | 0.21 | 0.0010 |

MS = mean squares; BE = bending energy; DA = directional asymmetry; FA = fluctuating asymmetry; R^2^ = coefficient of determination.
