# Supplementary material for: Morphological allometry constrains symmetric shape variation, but not asymmetry, of Halimeda tuna (Bryopsidales, Ulvophyceae) segments
Source: PLoS One. 2018 Oct 25;13(10):e0206492. doi: 10.1371/journal.pone.0206492 (PMC6201959; doi:10.1371/journal.pone.0206492)
Supplement: S6 Table — (DOC) [file pone.0206492.s007.doc]

**S6 Table. Multivariate Procrustes ANOVA models based on the configurations obtained from unslid equidistant semilandmarks, which decomposed symmetric variation and the components of asymmetry at the level of individual plants.**

| **Locality A, plant 01** | | | | | |
| --- | --- | --- | --- | --- | --- |
| **Source of variation** | **df** | **SS** | **MS** | **R^2^** | **F** |
| Segment | 14 | 0.5119 | 0.0366 | 0.749 | 3.552 |
| Side | 1 | 0.0103 | 0.0103 | 0.015 | 0.996 |
| Segment×Side | 14 | 0.1441 | 0.0103 | 0.211 | 18.289 |
| Measurement error | 30 | 0.0169 | 0.0006 | 0.025 |  |
| **Locality A, plant 02** | | | | | |
| **Source of variation** | **df** | **SS** | **MS** | **R^2^** | **F** |
| Segment | 5 | 0.1502 | 0.0300 | 0.849 | 6.696 |
| Side | 1 | 0.0014 | 0.0014 | 0.008 | 0.306 |
| Segment×Side | 5 | 0.0224 | 0.0045 | 0.127 | 19.152 |
| Measurement error | 12 | 0.0028 | 0.0002 | 0.016 |  |
| **Locality A, plant 03** | | | | | |
| **Source of variation** | **df** | **SS** | **MS** | **R^2^** | **F** |
| Segment | 5 | 0.1978 | 0.0396 | 0.595 | 3.905 |
| Side | 1 | 0.0774 | 0.0774 | 0.233 | 7.639 |
| Segment×Side | 5 | 0.0506 | 0.0101 | 0.152 | 18.919 |
| Measurement error | 12 | 0.0064 | 0.0005 | 0.019 |  |
| **Locality A, plant 04** | | | | | |
| **Source of variation** | **df** | **SS** | **MS** | **R^2^** | **F** |
| Segment | 11 | 0.1685 | 0.0153 | 0.532 | 1.407 |
| Side | 1 | 0.0122 | 0.0122 | 0.039 | 1.124 |
| Segment×Side | 11 | 0.1198 | 0.0109 | 0.378 | 16.038 |
| Measurement error | 24 | 0.0163 | 0.0007 | 0.051 |  |
| **Locality A, plant 05** | | | | | |
| **Source of variation** | **df** | **SS** | **MS** | **R^2^** | **F** |
| Segment | 7 | 0.1279 | 0.0183 | 0.853 | 9.804 |
| Side | 1 | 0.0039 | 0.0039 | 0.026 | 2.079 |
| Segment×Side | 7 | 0.0131 | 0.0019 | 0.087 | 5.845 |
| Measurement error | 16 | 0.0051 | 0.0003 | 0.034 |  |
| **Locality A, plant 06** | | | | | |
| **Source of variation** | **df** | **SS** | **MS** | **R^2^** | **F** |
| Segment | 9 | 0.4535 | 0.0503 | 0.886 | 11.035 |
| Side | 1 | 0.0077 | 0.0077 | 0.015 | 1.679 |
| Segment×Side | 9 | 0.0411 | 0.0046 | 0.080 | 9.804 |
| Measurement error | 20 | 0.0093 | 0.0005 | 0.018 |  |
| **Locality A, plant 07** | | | | | |
| **Source of variation** | **df** | **SS** | **MS** | **R^2^** | **F** |
| Segment | 7 | 0.1654 | 0.0236 | 0.891 | 10.371 |
| Side | 1 | 0.0010 | 0.0010 | 0.005 | 0.428 |
| Segment×Side | 7 | 0.0159 | 0.0023 | 0.086 | 10.799 |
| Measurement error | 16 | 0.0034 | 0.0002 | 0.018 |  |
| **Locality A, plant 08** | | | | | |
| **Source of variation** | **df** | **SS** | **MS** | **R^2^** | **F** |
| Segment | 6 | 0.3976 | 0.0663 | 0.729 | 3.590 |
| Side | 1 | 0.0247 | 0.0247 | 0.045 | 1.338 |
| Segment×Side | 6 | 0.1108 | 0.0185 | 0.203 | 22.214 |
| Measurement error | 14 | 0.0116 | 0.0008 | 0.021 |  |
| **Locality A, plant 09** | | | | | |
| **Source of variation** | **df** | **SS** | **MS** | **R^2^** | **F** |
| Segment | 6 | 0.1542 | 0.0257 | 0.897 | 11.738 |
| Side | 1 | 0.0028 | 0.0028 | 0.016 | 1.267 |
| Segment×Side | 6 | 0.0131 | 0.0022 | 0.076 | 17.927 |
| Measurement error | 14 | 0.0017 | 0.0001 | 0.010 |  |
| **Locality A, plant 10** | | | | | |
| **Source of variation** | **df** | **SS** | **MS** | **R^2^** | **F** |
| Segment | 9 | 0.2217 | 0.0246 | 0.796 | 4.596 |
| Side | 1 | 0.0059 | 0.0059 | 0.021 | 1.106 |
| Segment×Side | 9 | 0.0482 | 0.0054 | 0.173 | 38.665 |
| Measurement error | 20 | 0.0028 | 0.0001 | 0.010 |  |
| **Locality A, plant 11** | | | | | |
| **Source of variation** | **df** | **SS** | **MS** | **R^2^** | **F** |
| Segment | 11 | 0.1398 | 0.0127 | 0.757 | 3.539 |
| Side | 1 | 0.0006 | 0.0006 | 0.003 | 0.159 |
| Segment×Side | 11 | 0.0395 | 0.0036 | 0.214 | 17.938 |
| Measurement error | 24 | 0.0048 | 0.0002 | 0.026 |  |
| **Locality A, plant 12** | | | | | |
| **Source of variation** | **df** | **SS** | **MS** | **R^2^** | **F** |
| Segment | 8 | 0.1952 | 0.0244 | 0.792 | 4.529 |
| Side | 1 | 0.0046 | 0.0046 | 0.019 | 0.854 |
| Segment×Side | 8 | 0.0431 | 0.0054 | 0.175 | 26.591 |
| Measurement error | 18 | 0.0036 | 0.0002 | 0.015 |  |
| **Locality A, plant 13** | | | | | |
| **Source of variation** | **df** | **SS** | **MS** | **R^2^** | **F** |
| Segment | 14 | 0.3886 | 0.0278 | 0.869 | 7.557 |
| Side | 1 | 0.0009 | 0.0009 | 0.002 | 0.255 |
| Segment×Side | 14 | 0.0514 | 0.0037 | 0.115 | 19.106 |
| Measurement error | 30 | 0.0058 | 0.0002 | 0.013 |  |
| **Locality A, plant 14** | | | | | |
| **Source of variation** | **df** | **SS** | **MS** | **R^2^** | **F** |
| Segment | 9 | 0.2071 | 0.0230 | 0.773 | 5.905 |
| Side | 1 | 0.0059 | 0.0059 | 0.022 | 1.503 |
| Segment×Side | 9 | 0.0351 | 0.0039 | 0.131 | 3.898 |
| Measurement error | 20 | 0.0199 | 0.0010 | 0.075 |  |
| **Locality A, plant 15** | | | | | |
| **Source of variation** | **df** | **SS** | **MS** | **R^2^** | **F** |
| Segment | 10 | 0.1938 | 0.0194 | 0.803 | 5.291 |
| Side | 1 | 0.0014 | 0.0014 | 0.006 | 0.385 |
| Segment×Side | 10 | 0.0367 | 0.0037 | 0.152 | 8.603 |
| Measurement error | 22 | 0.0094 | 0.0004 | 0.039 |  |
| **Locality A, plant 16** | | | | | |
| **Source of variation** | **df** | **SS** | **MS** | **R^2^** | **F** |
| Segment | 8 | 0.3618 | 0.0452 | 0.820 | 5.513 |
| Side | 1 | 0.0029 | 0.0029 | 0.006 | 0.348 |
| Segment×Side | 8 | 0.0656 | 0.0082 | 0.149 | 13.582 |
| Measurement error | 18 | 0.0109 | 0.0006 | 0.025 |  |
| **Locality A, plant 17** | | | | | |
| **Source of variation** | **df** | **SS** | **MS** | **R^2^** | **F** |
| Segment | 6 | 0.1697 | 0.0283 | 0.851 | 7.265 |
| Side | 1 | 0.0036 | 0.0036 | 0.018 | 0.935 |
| Segment×Side | 6 | 0.0234 | 0.0039 | 0.117 | 20.583 |
| Measurement error | 14 | 0.0026 | 0.0002 | 0.013 |  |
| **Locality A, plant 18** | | | | | |
| **Source of variation** | **df** | **SS** | **MS** | **R^2^** | **F** |
| Segment | 15 | 0.2038 | 0.0136 | 0.648 | 2.076 |
| Side | 1 | 0.0015 | 0.0015 | 0.005 | 0.229 |
| Segment×Side | 15 | 0.0982 | 0.0065 | 0.312 | 18.665 |
| Measurement error | 32 | 0.0112 | 0.0004 | 0.036 |  |
| **Locality A, plant 19** | | | | | |
| **Source of variation** | **df** | **SS** | **MS** | **R^2^** | **F** |
| Segment | 8 | 0.2185 | 0.0273 | 0.871 | 7.777 |
| Side | 1 | 0.0015 | 0.0015 | 0.006 | 0.421 |
| Segment×Side | 8 | 0.0281 | 0.0035 | 0.112 | 23.212 |
| Measurement error | 18 | 0.0027 | 0.0002 | 0.011 |  |
| **Locality A, plant 20** | | | | | |
| **Source of variation** | **df** | **SS** | **MS** | **R^2^** | **F** |
| Segment | 10 | 0.1992 | 0.0199 | 0.751 | 3.738 |
| Side | 1 | 0.0045 | 0.0045 | 0.017 | 0.843 |
| Segment×Side | 10 | 0.0532 | 0.0053 | 0.201 | 14.181 |
| Measurement error | 22 | 0.0083 | 0.0004 | 0.031 |  |
| **Locality A, plant 21** | | | | | |
| **Source of variation** | **df** | **SS** | **MS** | **R^2^** | **F** |
| Segment | 7 | 0.1794 | 0.0256 | 0.795 | 4.816 |
| Side | 1 | 0.0016 | 0.0016 | 0.007 | 0.305 |
| Segment×Side | 7 | 0.0373 | 0.0053 | 0.165 | 11.338 |
| Measurement error | 16 | 0.0075 | 0.0005 | 0.033 |  |
| **Locality A, plant 22** | | | | | |
| **Source of variation** | **df** | **SS** | **MS** | **R^2^** | **F** |
| Segment | 8 | 0.2074 | 0.0259 | 0.759 | 5.026 |
| Side | 1 | 0.0132 | 0.0132 | 0.048 | 2.557 |
| Segment×Side | 8 | 0.0413 | 0.0052 | 0.151 | 8.406 |
| Measurement error | 18 | 0.0110 | 0.0006 | 0.040 |  |
| **Locality A, plant 23** | | | | | |
| **Source of variation** | **df** | **SS** | **MS** | **R^2^** | **F** |
| Segment | 9 | 0.3459 | 0.0384 | 0.777 | 4.333 |
| Side | 1 | 0.0107 | 0.0107 | 0.024 | 1.210 |
| Segment×Side | 9 | 0.0798 | 0.0089 | 0.179 | 19.738 |
| Measurement error | 20 | 0.0089 | 0.0004 | 0.020 |  |
| **Locality A, plant 24** | | | | | |
| **Source of variation** | **df** | **SS** | **MS** | **R^2^** | **F** |
| Segment | 9 | 0.1383 | 0.0154 | 0.733 | 3.508 |
| Side | 1 | 0.0018 | 0.0018 | 0.009 | 0.404 |
| Segment×Side | 9 | 0.0394 | 0.0044 | 0.209 | 9.546 |
| Measurement error | 20 | 0.0092 | 0.0005 | 0.049 |  |
| **Locality A, plant 25** | | | | | |
| **Source of variation** | **df** | **SS** | **MS** | **R^2^** | **F** |
| Segment | 10 | 0.2782 | 0.0278 | 0.610 | 2.165 |
| Side | 1 | 0.0319 | 0.0319 | 0.069 | 2.479 |
| Segment×Side | 10 | 0.1285 | 0.0129 | 0.282 | 16.343 |
| Measurement error | 22 | 0.0173 | 0.0008 | 0.038 |  |
| **Locality A, plant 26** | | | | | |
| **Source of variation** | **df** | **SS** | **MS** | **R^2^** | **F** |
| Segment | 8 | 0.3437 | 0.0429 | 0.865 | 7.571 |
| Side | 1 | 0.0042 | 0.0042 | 0.011 | 0.736 |
| Segment×Side | 8 | 0.0454 | 0.0057 | 0.114 | 25.703 |
| Measurement error | 18 | 0.0040 | 0.0002 | 0.010 |  |
| **Locality A, plant 27** | | | | | |
| **Source of variation** | **df** | **SS** | **MS** | **R^2^** | **F** |
| Segment | 7 | 0.2778 | 0.0397 | 0.769 | 5.825 |
| Side | 1 | 0.0207 | 0.0207 | 0.057 | 3.034 |
| Segment×Side | 7 | 0.0477 | 0.0068 | 0.132 | 7.456 |
| Measurement error | 16 | 0.0146 | 0.0009 | 0.041 |  |
| **Locality A, plant 28** | | | | | |
| **Source of variation** | **df** | **SS** | **MS** | **R^2^** | **F** |
| Segment | 9 | 0.2279 | 0.0253 | 0.777 | 4.449 |
| Side | 1 | 0.0053 | 0.0053 | 0.018 | 0.927 |
| Segment×Side | 9 | 0.0512 | 0.0057 | 0.175 | 12.536 |
| Measurement error | 20 | 0.0091 | 0.0005 | 0.031 |  |
| **Locality A, plant 29** | | | | | |
| **Source of variation** | **df** | **SS** | **MS** | **R^2^** | **F** |
| Segment | 9 | 0.0673 | 0.0075 | 0.655 | 2.342 |
| Side | 1 | 0.0007 | 0.0007 | 0.007 | 0.209 |
| Segment×Side | 9 | 0.0288 | 0.0032 | 0.279 | 10.531 |
| Measurement error | 20 | 0.0061 | 0.0003 | 0.059 |  |
| **Locality A, plant 30** | | | | | |
| **Source of variation** | **df** | **SS** | **MS** | **R^2^** | **F** |
| Segment | 11 | 0.3041 | 0.0276 | 0.907 | 13.665 |
| Side | 1 | 0.0023 | 0.0023 | 0.007 | 1.153 |
| Segment×Side | 11 | 0.0223 | 0.0020 | 0.066 | 7.478 |
| Measurement error | 24 | 0.0065 | 0.0003 | 0.019 |  |
| **Locality A, plant 31** | | | | | |
| **Source of variation** | **df** | **SS** | **MS** | **R^2^** | **F** |
| Segment | 6 | 0.1148 | 0.0191 | 0.843 | 9.324 |
| Side | 1 | 0.0078 | 0.0078 | 0.057 | 3.787 |
| Segment×Side | 6 | 0.0123 | 0.0021 | 0.090 | 20.926 |
| Measurement error | 14 | 0.0014 | 0.0001 | 0.010 |  |
| **Locality A, plant 32** | | | | | |
| **Source of variation** | **df** | **SS** | **MS** | **R^2^** | **F** |
| Segment | 12 | 0.0718 | 0.0059 | 0.556 | 1.471 |
| Side | 1 | 0.0049 | 0.0049 | 0.038 | 1.219 |
| Segment×Side | 12 | 0.0488 | 0.0041 | 0.378 | 28.925 |
| Measurement error | 26 | 0.0037 | 0.0001 | 0.028 |  |
| **Locality A, plant 33** | | | | | |
| **Source of variation** | **df** | **SS** | **MS** | **R^2^** | **F** |
| Segment | 6 | 0.3020 | 0.0503 | 0.723 | 2.882 |
| Side | 1 | 0.0069 | 0.0069 | 0.166 | 0.398 |
| Segment×Side | 6 | 0.1047 | 0.0174 | 0.251 | 57.855 |
| Measurement error | 14 | 0.0042 | 0.0003 | 0.010 |  |
| **Locality A, plant 34** | | | | | |
| **Source of variation** | **df** | **SS** | **MS** | **R^2^** | **F** |
| Segment | 6 | 0.0370 | 0.0062 | 0.586 | 1.825 |
| Side | 1 | 0.0047 | 0.0047 | 0.074 | 1.377 |
| Segment×Side | 6 | 0.0203 | 0.0034 | 0.321 | 39.209 |
| Measurement error | 14 | 0.0012 | 0.0001 | 0.019 |  |
| **Locality A, plant 35** | | | | | |
| **Source of variation** | **df** | **SS** | **MS** | **R^2^** | **F** |
| Segment | 5 | 0.2309 | 0.0461 | 0.945 | 28.141 |
| Side | 1 | 0.0042 | 0.0042 | 0.017 | 2.559 |
| Segment×Side | 5 | 0.0082 | 0.0016 | 0.034 | 21.497 |
| Measurement error | 12 | 0.0009 | 0.0001 | 0.004 |  |
| **Locality A, plant 36** | | | | | |
| **Source of variation** | **df** | **SS** | **MS** | **R^2^** | **F** |
| Segment | 16 | 0.4038 | 0.0252 | 0.805 | 5.379 |
| Side | 1 | 0.0029 | 0.0029 | 0.006 | 0.631 |
| Segment×Side | 16 | 0.0751 | 0.0047 | 0.149 | 7.972 |
| Measurement error | 34 | 0.0200 | 0.0006 | 0.039 |  |
| **Locality A, plant 37** | | | | | |
| **Source of variation** | **df** | **SS** | **MS** | **R^2^** | **F** |
| Segment | 7 | 0.0574 | 0.0082 | 0.691 | 3.348 |
| Side | 1 | 0.0051 | 0.0051 | 0.062 | 2.093 |
| Segment×Side | 7 | 0.0171 | 0.0024 | 0.206 | 11.616 |
| Measurement error | 16 | 0.0034 | 0.0002 | 0.041 |  |
| **Locality A, plant 38** | | | | | |
| **Source of variation** | **df** | **SS** | **MS** | **R^2^** | **F** |
| Segment | 6 | 0.2472 | 0.0412 | 0.846 | 7.637 |
| Side | 1 | 0.0069 | 0.0069 | 0.024 | 1.284 |
| Segment×Side | 6 | 0.0324 | 0.0054 | 0.111 | 13.069 |
| Measurement error | 14 | 0.0058 | 0.0004 | 0.020 |  |
| **Locality A, plant 39** | | | | | |
| **Source of variation** | **df** | **SS** | **MS** | **R^2^** | **F** |
| Segment | 5 | 0.0652 | 0.0130 | 0.741 | 5.616 |
| Side | 1 | 0.0092 | 0.0092 | 0.104 | 3.944 |
| Segment×Side | 5 | 0.0116 | 0.0023 | 0.132 | 13.940 |
| Measurement error | 12 | 0.0019 | 0.0002 | 0.023 |  |
| **Locality A, plant 40** | | | | | |
| **Source of variation** | **df** | **SS** | **MS** | **R^2^** | **F** |
| Segment | 8 | 0.1466 | 0.0183 | 0.767 | 3.801 |
| Side | 1 | 0.0034 | 0.0034 | 0.018 | 0.702 |
| Segment×Side | 8 | 0.0386 | 0.0048 | 0.202 | 34.931 |
| Measurement error | 18 | 0.0025 | 0.0001 | 0.013 |  |
| **Locality A, plant 41** | | | | | |
| **Source of variation** | **df** | **SS** | **MS** | **R^2^** | **F** |
| Segment | 11 | 0.2358 | 0.0214 | 0.771 | 3.804 |
| Side | 1 | 0.0026 | 0.0026 | 0.008 | 0.457 |
| Segment×Side | 11 | 0.0619 | 0.0056 | 0.203 | 25.113 |
| Measurement error | 24 | 0.0054 | 0.0002 | 0.018 |  |
| **Locality A, plant 42** | | | | | |
| **Source of variation** | **df** | **SS** | **MS** | **R^2^** | **F** |
| Segment | 12 | 0.2774 | 0.0231 | 0.772 | 4.156 |
| Side | 1 | 0.0021 | 0.0021 | 0.006 | 0.382 |
| Segment×Side | 12 | 0.0667 | 0.0056 | 0.186 | 10.899 |
| Measurement error | 26 | 0.0133 | 0.0005 | 0.037 |  |
| **Locality A, plant 43** | | | | | |
| **Source of variation** | **df** | **SS** | **MS** | **R^2^** | **F** |
| Segment | 14 | 0.3044 | 0.0217 | 0.784 | 5.095 |
| Side | 1 | 0.0135 | 0.0135 | 0.035 | 3.166 |
| Segment×Side | 14 | 0.0597 | 0.0043 | 0.154 | 12.306 |
| Measurement error | 30 | 0.0104 | 0.0003 | 0.027 |  |
| **Locality A, plant 44** | | | | | |
| **Source of variation** | **df** | **SS** | **MS** | **R^2^** | **F** |
| Segment | 9 | 0.2399 | 0.0267 | 0.778 | 4.279 |
| Side | 1 | 0.0072 | 0.0072 | 0.023 | 1.162 |
| Segment×Side | 9 | 0.0561 | 0.0062 | 0.182 | 23.404 |
| Measurement error | 20 | 0.0053 | 0.0003 | 0.017 |  |
| **Locality A, plant 45** | | | | | |
| **Source of variation** | **df** | **SS** | **MS** | **R^2^** | **F** |
| Segment | 16 | 0.5499 | 0.0344 | 0.856 | 8.663 |
| Side | 1 | 0.0028 | 0.0028 | 0.004 | 0.713 |
| Segment×Side | 16 | 0.0635 | 0.0039 | 0.099 | 5.138 |
| Measurement error | 34 | 0.0263 | 0.0008 | 0.041 |  |
| **Locality A, plant 46** | | | | | |
| **Source of variation** | **df** | **SS** | **MS** | **R^2^** | **F** |
| Segment | 8 | 0.2466 | 0.0308 | 0.724 | 2.768 |
| Side | 1 | 0.0007 | 0.0007 | 0.002 | 0.066 |
| Segment×Side | 8 | 0.0891 | 0.0111 | 0.261 | 47.437 |
| Measurement error | 18 | 0.0042 | 0.0002 | 0.012 |  |
| **Locality A, plant 47** | | | | | |
| **Source of variation** | **df** | **SS** | **MS** | **R^2^** | **F** |
| Segment | 9 | 0.1392 | 0.0155 | 0.682 | 2.754 |
| Side | 1 | 0.0051 | 0.0051 | 0.025 | 0.912 |
| Segment×Side | 9 | 0.0505 | 0.0056 | 0.248 | 12.072 |
| Measurement error | 20 | 0.0093 | 0.0005 | 0.046 |  |
| **Locality A, plant 48** | | | | | |
| **Source of variation** | **df** | **SS** | **MS** | **R^2^** | **F** |
| Segment | 7 | 0.0784 | 0.0112 | 0.634 | 2.401 |
| Side | 1 | 0.0097 | 0.0097 | 0.079 | 2.091 |
| Segment×Side | 7 | 0.0326 | 0.0047 | 0.264 | 26.032 |
| Measurement error | 16 | 0.0029 | 0.0002 | 0.023 |  |
| **Locality B, plant 01** | | | | | |
| **Source of variation** | **df** | **SS** | **MS** | **R^2^** | **F** |
| Segment | 9 | 0.2169 | 0.0241 | 0.812 | 7.999 |
| Side | 1 | 0.0032 | 0.0032 | 0.012 | 1.065 |
| Segment×Side | 9 | 0.0271 | 0.0030 | 0.101 | 3.022 |
| Measurement error | 20 | 0.0199 | 0.0010 | 0.075 |  |
| **Locality B, plant 02** | | | | | |
| **Source of variation** | **df** | **SS** | **MS** | **R^2^** | **F** |
| Segment | 8 | 0.2755 | 0.0344 | 0.837 | 9.248 |
| Side | 1 | 0.0067 | 0.0067 | 0.021 | 1.818 |
| Segment×Side | 8 | 0.0298 | 0.0037 | 0.090 | 3.889 |
| Measurement error | 18 | 0.0172 | 0.0009 | 0.052 |  |
| **Locality B, plant 03** | | | | | |
| **Source of variation** | **df** | **SS** | **MS** | **R^2^** | **F** |
| Segment | 9 | 0.3663 | 0.0407 | 0.898 | 13.527 |
| Side | 1 | 0.0022 | 0.0022 | 0.005 | 0.737 |
| Segment×Side | 9 | 0.0271 | 0.0030 | 0.066 | 4.801 |
| Measurement error | 20 | 0.0125 | 0.0006 | 0.031 |  |
| **Locality B, plant 04** | | | | | |
| **Source of variation** | **df** | **SS** | **MS** | **R^2^** | **F** |
| Segment | 9 | 0.2920 | 0.0324 | 0.825 | 5.673 |
| Side | 1 | 0.0023 | 0.0023 | 0.006 | 0.401 |
| Segment×Side | 9 | 0.0515 | 0.0057 | 0.146 | 14.365 |
| Measurement error | 20 | 0.0079 | 0.0004 | 0.023 |  |
| **Locality B, plant 05** | | | | | |
| **Source of variation** | **df** | **SS** | **MS** | **R^2^** | **F** |
| Segment | 7 | 0.1308 | 0.0187 | 0.694 | 3.063 |
| Side | 1 | 0.0116 | 0.0116 | 0.062 | 1.903 |
| Segment×Side | 7 | 0.0427 | 0.0061 | 0.227 | 30.194 |
| Measurement error | 16 | 0.0032 | 0.0002 | 0.017 |  |
| **Locality B, plant 06** | | | | | |
| **Source of variation** | **df** | **SS** | **MS** | **R^2^** | **F** |
| Segment | 7 | 0.1753 | 0.0250 | 0.842 | 6.639 |
| Side | 1 | 0.0018 | 0.0018 | 0.009 | 0.487 |
| Segment×Side | 7 | 0.0264 | 0.0038 | 0.127 | 13.033 |
| Measurement error | 16 | 0.0046 | 0.0003 | 0.022 |  |
| **Locality B, plant 07** | | | | | |
| **Source of variation** | **df** | **SS** | **MS** | **R^2^** | **F** |
| Segment | 8 | 0.1459 | 0.0182 | 0.829 | 6.044 |
| Side | 1 | 0.0029 | 0.0029 | 0.017 | 0.972 |
| Segment×Side | 8 | 0.0241 | 0.0030 | 0.137 | 17.442 |
| Measurement error | 18 | 0.0031 | 0.0002 | 0.018 |  |
| **Locality B, plant 08** | | | | | |
| **Source of variation** | **df** | **SS** | **MS** | **R^2^** | **F** |
| Segment | 7 | 0.1178 | 0.0168 | 0.900 | 17.075 |
| Side | 1 | 0.0028 | 0.0028 | 0.022 | 2.857 |
| Segment×Side | 7 | 0.0069 | 0.0010 | 0.053 | 4.704 |
| Measurement error | 16 | 0.0034 | 0.0002 | 0.026 |  |
| **Locality B, plant 09** | | | | | |
| **Source of variation** | **df** | **SS** | **MS** | **R^2^** | **F** |
| Segment | 9 | 0.1675 | 0.0186 | 0.883 | 8.872 |
| Side | 1 | 0.0008 | 0.0008 | 0.004 | 0.368 |
| Segment×Side | 9 | 0.0189 | 0.0021 | 0.099 | 17.182 |
| Measurement error | 20 | 0.0024 | 0.0001 | 0.013 |  |
| **Locality B, plant 10** | | | | | |
| **Source of variation** | **df** | **SS** | **MS** | **R^2^** | **F** |
| Segment | 7 | 0.2124 | 0.0303 | 0.856 | 6.769 |
| Side | 1 | 0.0015 | 0.0015 | 0.006 | 0.333 |
| Segment×Side | 7 | 0.0314 | 0.0045 | 0.126 | 25.523 |
| Measurement error | 16 | 0.0028 | 0.0002 | 0.011 |  |
| **Locality B, plant 11** | | | | | |
| **Source of variation** | **df** | **SS** | **MS** | **R^2^** | **F** |
| Segment | 8 | 0.1402 | 0.0175 | 0.657 | 2.373 |
| Side | 1 | 0.0037 | 0.0037 | 0.017 | 0.498 |
| Segment×Side | 8 | 0.0591 | 0.0074 | 0.277 | 13.022 |
| Measurement error | 18 | 0.0102 | 0.0006 | 0.048 |  |
| **Locality B, plant 12** | | | | | |
| **Source of variation** | **df** | **SS** | **MS** | **R^2^** | **F** |
| Segment | 12 | 0.1556 | 0.0129 | 0.607 | 1.905 |
| Side | 1 | 0.0087 | 0.0087 | 0.034 | 1.274 |
| Segment×Side | 12 | 0.0817 | 0.0068 | 0.318 | 16.784 |
| Measurement error | 26 | 0.0105 | 0.0004 | 0.041 |  |
| **Locality B, plant 13** | | | | | |
| **Source of variation** | **df** | **SS** | **MS** | **R^2^** | **F** |
| Segment | 8 | 0.0761 | 0.0095 | 0.769 | 5.449 |
| Side | 1 | 0.0012 | 0.0012 | 0.012 | 0.688 |
| Segment×Side | 8 | 0.0139 | 0.0017 | 0.141 | 4.124 |
| Measurement error | 18 | 0.0076 | 0.0004 | 0.077 |  |
| **Locality B, plant 14** | | | | | |
| **Source of variation** | **df** | **SS** | **MS** | **R^2^** | **F** |
| Segment | 15 | 0.3621 | 0.0241 | 0.874 | 8.984 |
| Side | 1 | 0.0033 | 0.0033 | 0.008 | 1.247 |
| Segment×Side | 15 | 0.0403 | 0.0027 | 0.097 | 10.139 |
| Measurement error | 32 | 0.0084 | 0.0003 | 0.020 |  |
| **Locality B, plant 15** | | | | | |
| **Source of variation** | **df** | **SS** | **MS** | **R^2^** | **F** |
| Segment | 14 | 0.4051 | 0.0289 | 0.918 | 20.519 |
| Side | 1 | 0.0075 | 0.0075 | 0.017 | 5.332 |
| Segment×Side | 14 | 0.0197 | 0.0014 | 0.045 | 4.814 |
| Measurement error | 30 | 0.0088 | 0.0003 | 0.020 |  |
| **Locality B, plant 16** | | | | | |
| **Source of variation** | **df** | **SS** | **MS** | **R^2^** | **F** |
| Segment | 6 | 0.0740 | 0.0123 | 0.824 | 5.466 |
| Side | 1 | 0.0009 | 0.0009 | 0.009 | 0.378 |
| Segment×Side | 6 | 0.0135 | 0.0023 | 0.151 | 21.941 |
| Measurement error | 14 | 0.0014 | 0.0001 | 0.016 |  |
| **Locality B, plant 17** | | | | | |
| **Source of variation** | **df** | **SS** | **MS** | **R^2^** | **F** |
| Segment | 23 | 0.4517 | 0.0196 | 0.704 | 2.588 |
| Side | 1 | 0.0011 | 0.0011 | 0.002 | 0.140 |
| Segment×Side | 23 | 0.1745 | 0.0076 | 0.272 | 25.262 |
| Measurement error | 48 | 0.0144 | 0.0003 | 0.022 |  |
| **Locality B, plant 18** | | | | | |
| **Source of variation** | **df** | **SS** | **MS** | **R^2^** | **F** |
| Segment | 8 | 0.2205 | 0.0276 | 0.872 | 9.678 |
| Side | 1 | 0.0053 | 0.0053 | 0.021 | 1.863 |
| Segment×Side | 8 | 0.0228 | 0.0028 | 0.090 | 11.635 |
| Measurement error | 18 | 0.0044 | 0.0002 | 0.017 |  |
| **Locality B, plant 19** | | | | | |
| **Source of variation** | **df** | **SS** | **MS** | **R^2^** | **F** |
| Segment | 13 | 0.2089 | 0.0161 | 0.801 | 4.684 |
| Side | 1 | 0.0021 | 0.0021 | 0.008 | 0.614 |
| Segment×Side | 13 | 0.0446 | 0.0034 | 0.171 | 18.533 |
| Measurement error | 28 | 0.0052 | 0.0002 | 0.020 |  |
| **Locality B, plant 20** | | | | | |
| **Source of variation** | **df** | **SS** | **MS** | **R^2^** | **F** |
| Segment | 21 | 0.3115 | 0.0148 | 0.789 | 4.412 |
| Side | 1 | 0.0005 | 0.0005 | 0.002 | 0.142 |
| Segment×Side | 21 | 0.0706 | 0.0034 | 0.179 | 12.419 |
| Measurement error | 44 | 0.0119 | 0.0003 | 0.030 |  |
| **Locality B, plant 21** | | | | | |
| **Source of variation** | **df** | **SS** | **MS** | **R^2^** | **F** |
| Segment | 9 | 0.0948 | 0.0105 | 0.778 | 4.203 |
| Side | 1 | 0.0012 | 0.0012 | 0.010 | 0.466 |
| Segment×Side | 9 | 0.0225 | 0.0025 | 0.185 | 14.989 |
| Measurement error | 20 | 0.0033 | 0.0002 | 0.027 |  |
| **Locality B, plant 22** | | | | | |
| **Source of variation** | **df** | **SS** | **MS** | **R^2^** | **F** |
| Segment | 7 | 0.0753 | 0.0108 | 0.908 | 13.312 |
| Side | 1 | 0.0006 | 0.0006 | 0.007 | 0.685 |
| Segment×Side | 7 | 0.0057 | 0.0008 | 0.068 | 9.411 |
| Measurement error | 16 | 0.0014 | 0.0001 | 0.017 |  |
| **Locality B, plant 23** | | | | | |
| **Source of variation** | **df** | **SS** | **MS** | **R^2^** | **F** |
| Segment | 17 | 0.2474 | 0.0146 | 0.838 | 6.889 |
| Side | 1 | 0.0028 | 0.0028 | 0.009 | 1.348 |
| Segment×Side | 17 | 0.0359 | 0.0021 | 0.122 | 8.393 |
| Measurement error | 36 | 0.0091 | 0.0003 | 0.031 |  |
| **Locality B, plant 24** | | | | | |
| **Source of variation** | **df** | **SS** | **MS** | **R^2^** | **F** |
| Segment | 14 | 0.1232 | 0.0088 | 0.796 | 5.275 |
| Side | 1 | 0.0027 | 0.0027 | 0.018 | 1.643 |
| Segment×Side | 14 | 0.0233 | 0.0017 | 0.151 | 9.220 |
| Measurement error | 30 | 0.0054 | 0.0002 | 0.035 |  |
| **Locality B, plant 25** | | | | | |
| **Source of variation** | **df** | **SS** | **MS** | **R^2^** | **F** |
| Segment | 8 | 0.1422 | 0.0178 | 0.882 | 10.271 |
| Side | 1 | 0.0016 | 0.0016 | 0.01 | 0.906 |
| Segment×Side | 8 | 0.0138 | 0.0017 | 0.086 | 8.414 |
| Measurement error | 18 | 0.0037 | 0.0002 | 0.023 |  |
| **Locality B, plant 26** | | | | | |
| **Source of variation** | **df** | **SS** | **MS** | **R^2^** | **F** |
| Segment | 9 | 0.0228 | 0.0025 | 0.478 | 1.198 |
| Side | 1 | 0.0027 | 0.0027 | 0.057 | 1.284 |
| Segment×Side | 9 | 0.0190 | 0.0021 | 0.399 | 13.554 |
| Measurement error | 20 | 0.0031 | 0.0002 | 0.065 |  |
| **Locality B, plant 27** | | | | | |
| **Source of variation** | **df** | **SS** | **MS** | **R^2^** | **F** |
| Segment | 7 | 0.2412 | 0.0345 | 0.899 | 10.686 |
| Side | 1 | 0.0025 | 0.0025 | 0.009 | 0.760 |
| Segment×Side | 7 | 0.0226 | 0.0032 | 0.084 | 26.877 |
| Measurement error | 16 | 0.0019 | 0.0001 | 0.007 |  |
| **Locality B, plant 28** | | | | | |
| **Source of variation** | **df** | **SS** | **MS** | **R^2^** | **F** |
| Segment | 5 | 0.1273 | 0.0255 | 0.911 | 20.184 |
| Side | 1 | 0.0042 | 0.0042 | 0.030 | 3.361 |
| Segment×Side | 5 | 0.0063 | 0.0013 | 0.045 | 8.275 |
| Measurement error | 12 | 0.0018 | 0.0002 | 0.013 |  |
| **Locality B, plant 29** | | | | | |
| **Source of variation** | **df** | **SS** | **MS** | **R^2^** | **F** |
| Segment | 6 | 0.0271 | 0.0045 | 0.602 | 2.104 |
| Side | 1 | 0.0027 | 0.0027 | 0.060 | 1.260 |
| Segment×Side | 6 | 0.0129 | 0.0021 | 0.286 | 12.894 |
| Measurement error | 14 | 0.0023 | 0.0002 | 0.052 |  |
| **Locality B, plant 30** | | | | | |
| **Source of variation** | **df** | **SS** | **MS** | **R^2^** | **F** |
| Segment | 9 | 0.0929 | 0.0103 | 0.754 | 4.369 |
| Side | 1 | 0.0011 | 0.0011 | 0.009 | 0.476 |
| Segment×Side | 9 | 0.0213 | 0.0024 | 0.173 | 5.971 |
| Measurement error | 20 | 0.0079 | 0.0004 | 0.064 |  |
| **Locality B, plant 31** | | | | | |
| **Source of variation** | **df** | **SS** | **MS** | **R^2^** | **F** |
| Segment | 7 | 0.1417 | 0.0202 | 0.708 | 3.006 |
| Side | 1 | 0.0081 | 0.0081 | 0.041 | 1.205 |
| Segment×Side | 7 | 0.0471 | 0.0067 | 0.235 | 33.182 |
| Measurement error | 16 | 0.0032 | 0.0002 | 0.016 |  |
| **Locality B, plant 32** | | | | | |
| **Source of variation** | **df** | **SS** | **MS** | **R^2^** | **F** |
| Segment | 7 | 0.1233 | 0.0176 | 0.712 | 3.816 |
| Side | 1 | 0.0159 | 0.0159 | 0.092 | 3.461 |
| Segment×Side | 7 | 0.0323 | 0.0046 | 0.187 | 44.908 |
| Measurement error | 16 | 0.0016 | 0.0001 | 0.009 |  |
| **Locality B, plant 33** | | | | | |
| **Source of variation** | **df** | **SS** | **MS** | **R^2^** | **F** |
| Segment | 11 | 0.1808 | 0.0164 | 0.672 | 2.179 |
| Side | 1 | 0.0013 | 0.0013 | 0.005 | 0.174 |
| Segment×Side | 11 | 0.0829 | 0.0075 | 0.308 | 45.149 |
| Measurement error | 24 | 0.0040 | 0.0002 | 0.015 |  |
| **Locality B, plant 34** | | | | | |
| **Source of variation** | **df** | **SS** | **MS** | **R^2^** | **F** |
| Segment | 7 | 0.1216 | 0.0174 | 0.897 | 11.435 |
| Side | 1 | 0.0014 | 0.0014 | 0.010 | 0.889 |
| Segment×Side | 7 | 0.0106 | 0.0015 | 0.078 | 12.127 |
| Measurement error | 16 | 0.0020 | 0.0001 | 0.015 |  |
| **Locality B, plant 35** | | | | | |
| **Source of variation** | **df** | **SS** | **MS** | **R^2^** | **F** |
| Segment | 8 | 0.1682 | 0.0210 | 0.913 | 21.878 |
| Side | 1 | 0.0065 | 0.0065 | 0.035 | 6.751 |
| Segment×Side | 8 | 0.0077 | 0.0009 | 0.042 | 9.092 |
| Measurement error | 18 | 0.0019 | 0.0001 | 0.010 |  |
| **Locality B, plant 36** | | | | | |
| **Source of variation** | **df** | **SS** | **MS** | **R^2^** | **F** |
| Segment | 6 | 0.1319 | 0.0219 | 0.895 | 11.316 |
| Side | 1 | 0.0023 | 0.0023 | 0.016 | 1.181 |
| Segment×Side | 6 | 0.0117 | 0.0019 | 0.079 | 18.641 |
| Measurement error | 14 | 0.0015 | 0.0001 | 0.010 |  |
| **Locality B, plant 37** | | | | | |
| **Source of variation** | **df** | **SS** | **MS** | **R^2^** | **F** |
| Segment | 9 | 0.0449 | 0.0050 | 0.706 | 3.668 |
| Side | 1 | 0.0007 | 0.0007 | 0.011 | 0.507 |
| Segment×Side | 9 | 0.0122 | 0.0014 | 0.192 | 4.684 |
| Measurement error | 20 | 0.0058 | 0.0003 | 0.091 |  |
| **Locality B, plant 38** | | | | | |
| **Source of variation** | **df** | **SS** | **MS** | **R^2^** | **F** |
| Segment | 8 | 0.1262 | 0.0158 | 0.766 | 3.746 |
| Side | 1 | 0.0029 | 0.0029 | 0.017 | 0.678 |
| Segment×Side | 8 | 0.0337 | 0.0042 | 0.205 | 39.518 |
| Measurement error | 18 | 0.0019 | 0.0001 | 0.012 |  |
| **Locality B, plant 39** | | | | | |
| **Source of variation** | **df** | **SS** | **MS** | **R^2^** | **F** |
| Segment | 8 | 0.0856 | 0.0107 | 0.709 | 2.681 |
| Side | 1 | 0.0011 | 0.0011 | 0.009 | 0.280 |
| Segment×Side | 8 | 0.0319 | 0.0040 | 0.264 | 34.892 |
| Measurement error | 18 | 0.0021 | 0.0001 | 0.017 |  |
| **Locality B, plant 40** | | | | | |
| **Source of variation** | **df** | **SS** | **MS** | **R^2^** | **F** |
| Segment | 9 | 0.0842 | 0.0094 | 0.790 | 4.228 |
| Side | 1 | 0.0005 | 0.0005 | 0.005 | 0.224 |
| Segment×Side | 9 | 0.0199 | 0.0022 | 0.187 | 22.858 |
| Measurement error | 20 | 0.0019 | 0.0001 | 0.018 |  |
| **Locality B, plant 41** | | | | | |
| **Source of variation** | **df** | **SS** | **MS** | **R^2^** | **F** |
| Segment | 10 | 0.1093 | 0.0109 | 0.789 | 6.008 |
| Side | 1 | 0.0075 | 0.0075 | 0.054 | 4.103 |
| Segment×Side | 10 | 0.0182 | 0.0018 | 0.131 | 11.072 |
| Measurement error | 22 | 0.0036 | 0.0002 | 0.026 |  |
| **Locality B, plant 42** | | | | | |
| **Source of variation** | **df** | **SS** | **MS** | **R^2^** | **F** |
| Segment | 6 | 0.1191 | 0.0199 | 0.781 | 5.841 |
| Side | 1 | 0.0109 | 0.0109 | 0.072 | 3.213 |
| Segment×Side | 6 | 0.0204 | 0.0034 | 0.134 | 22.446 |
| Measurement error | 14 | 0.0021 | 0.0002 | 0.014 |  |
| **Locality B, plant 43** | | | | | |
| **Source of variation** | **df** | **SS** | **MS** | **R^2^** | **F** |
| Segment | 11 | 0.2386 | 0.0217 | 0.899 | 11.845 |
| Side | 1 | 0.0015 | 0.0015 | 0.006 | 0.798 |
| Segment×Side | 11 | 0.0201 | 0.0018 | 0.076 | 8.494 |
| Measurement error | 24 | 0.0052 | 0.0002 | 0.019 |  |
| **Locality B, plant 44** | | | | | |
| **Source of variation** | **df** | **SS** | **MS** | **R^2^** | **F** |
| Segment | 13 | 0.3154 | 0.0243 | 0.904 | 12.601 |
| Side | 1 | 0.0038 | 0.0038 | 0.110 | 1.989 |
| Segment×Side | 13 | 0.0250 | 0.0019 | 0.072 | 12.118 |
| Measurement error | 28 | 0.0044 | 0.0002 | 0.013 |  |
| **Locality B, plant 45** | | | | | |
| **Source of variation** | **df** | **SS** | **MS** | **R^2^** | **F** |
| Segment | 11 | 0.1627 | 0.0148 | 0.814 | 5.806 |
| Side | 1 | 0.0037 | 0.0037 | 0.018 | 1.439 |
| Segment×Side | 11 | 0.0280 | 0.0025 | 0.140 | 10.996 |
| Measurement error | 24 | 0.0056 | 0.0002 | 0.028 |  |
| **Locality B, plant 46** | | | | | |
| **Source of variation** | **df** | **SS** | **MS** | **R^2^** | **F** |
| Segment | 6 | 0.0384 | 0.0064 | 0.595 | 2.535 |
| Side | 1 | 0.0053 | 0.0053 | 0.082 | 2.082 |
| Segment×Side | 6 | 0.0152 | 0.0025 | 0.235 | 6.213 |
| Measurement error | 14 | 0.0057 | 0.0004 | 0.088 |  |
| **Locality B, plant 47** | | | | | |
| **Source of variation** | **df** | **SS** | **MS** | **R^2^** | **F** |
| Segment | 10 | 0.1771 | 0.0177 | 0.682 | 2.935 |
| Side | 1 | 0.0035 | 0.0035 | 0.014 | 0.583 |
| Segment×Side | 10 | 0.0603 | 0.0060 | 0.233 | 7.213 |
| Measurement error | 22 | 0.0184 | 0.0008 | 0.071 |  |
| **Locality B, plant 48** | | | | | |
| **Source of variation** | **df** | **SS** | **MS** | **R^2^** | **F** |
| Segment | 12 | 0.2826 | 0.0235 | 0.862 | 7.739 |
| Side | 1 | 0.0033 | 0.0033 | 0.010 | 1.094 |
| Segment×Side | 12 | 0.0365 | 0.0030 | 0.111 | 14.924 |
| Measurement error | 26 | 0.0053 | 0.0002 | 0.016 |  |

df = degrees of freedom; SS = sums of squares; MS = mean squares, R^2^ = coefficient of determination.
